# Progressive Failure Analysis of Wellbore Cement Sheaths in Salt Cavern Energy Storage Under Gas Injection–Production Cycles

**DOI:** 10.1007/s00603-026-05313-5

**Published:** 2026-02-09

**Authors:** Xin Liu, Xuan Kang, Yadong Wang, Yunteng Wang, Wei Wu

**Affiliations:** Institut für Geotechnik, Universität für Bodenkultur Wien (BOKU University), Feistmantelstraße 4, 1190 Wien, Austria

**Keywords:** Salt cavern gas storage, Wellbore cement sheath, Gas thermodynamics, Damage and fracture evolution, Phase-field modeling

## Abstract

In this work, we propose a computational paradigm for gas thermo-mechanical coupling in salt cavern gas storage systems within the phase-field framework. The novelty lies in integrating a thermodynamic gas-flow model with the phase-field formulations to simultaneously capture the gas pressure, temperature evolution, and the deformation–failure behavior of the cement sheath. The validation against analytical solutions, laboratory tests, and field-scale monitoring data confirms the robustness of our approach. Our simulations reveal that the gas pressure varies nearly linearly during injection–production cycles, while the temperature evolves nonlinearly with localized hot and cold zones. The failure patterns are depth-dependent: tensile damage dominates near the wellhead, whereas mixed-mode failure occurs at greater depths, which is consistent with field observations. The thermal cycling drives progressive damage and reduces heat transfer capacity in the fractured zones. The parametric studies highlight the strong influence of the lateral stress coefficient and fracture energy on critical operating pressures. Progressive injection–production strategies are recommended to enhance wellbore integrity and optimize storage capacity.

## Highlights

- Thermodynamic gas-flow model integrated with phase-field formulations for coupled gas–thermal–mechanical simulations.
- We validate against analytical, experimental, and field data; and we reveal depth-dependent failure and gas nonlinear thermo-mechanical responses.
- Identifies controlling factors and recommends progressive cycling strategies to enhance wellbore integrity and storage capacity.

## Introduction

**Fig. 1:**
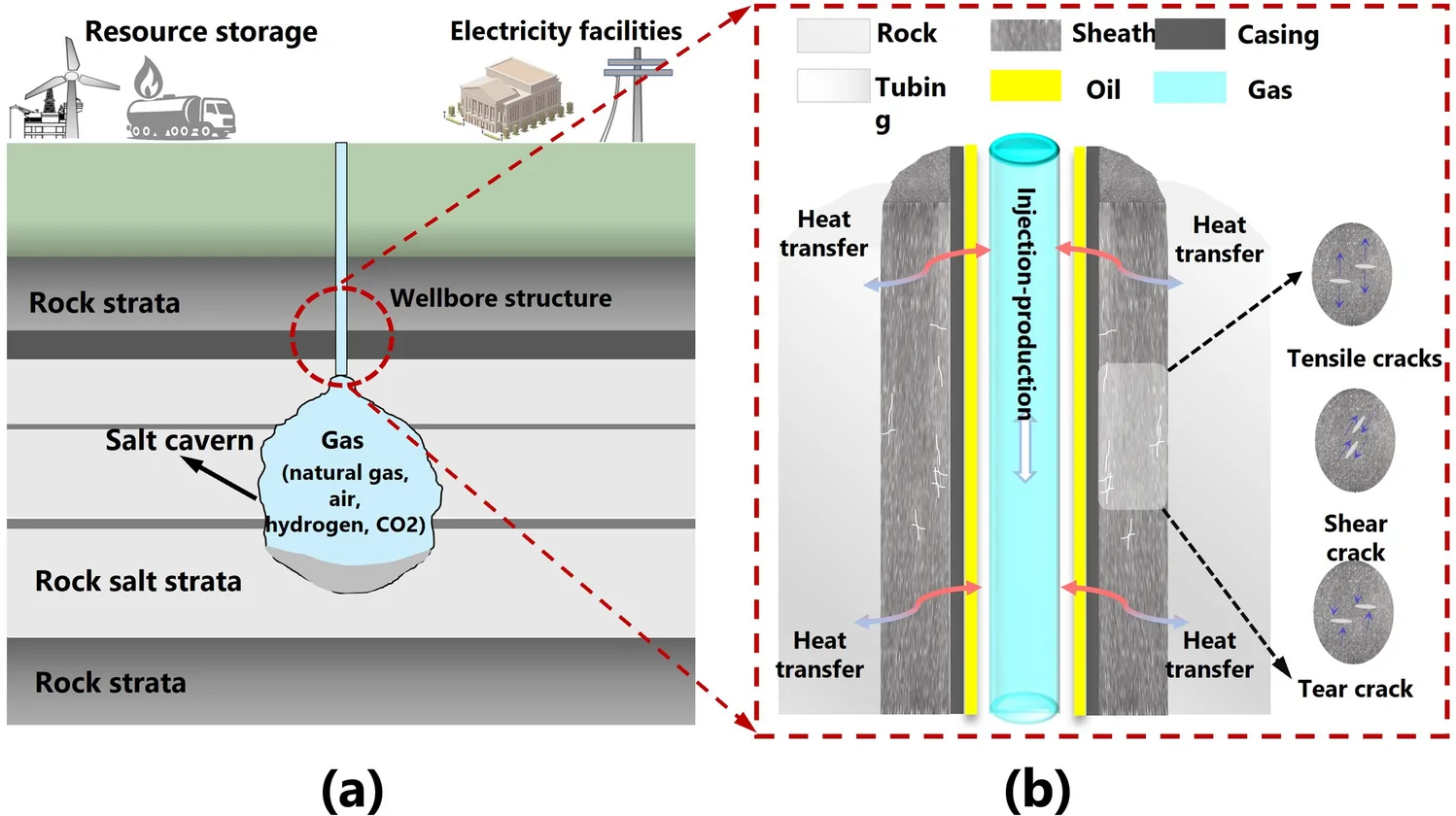
Schematics of **a** underground gas storage in a salt cavern and **b** enlarged wellbore structures under gas injection–production cycles in a thermo-mechanical coupling environment, showing different macroscopic cracks in the cement sheath

The excellent sealing performance, self-healing capability, and long-term stability of salt caverns make them a preferred geological setting for subsurface energy storage (Yang et al. 2015; Maraggi and Moscardelli 2024; Wei et al. 2025), which have been widely used to store natural gas, petroleum, carbon dioxide, hydrogen, and other substances, as shown in Fig. 1a. In recent years, the rise of renewable energy sources, such as wind power, photovoltaics, and hydrogen or helium, drives the rapid developments of subsurface energy storage systems in salt caverns (Wei et al. 2023; Zhu et al. 2023; Maraggi and Moscardelli 2024; Mwakipunda et al. 2025). In these storage systems, the operating environments are characterized by strong intermittency and high stochasticity due to the high-frequency and high-intensity injection–production cycles. These extreme operating conditions, e.g., high in-situ confining pressure, rapid and intense fluctuations in temperature (gradient), and variations in pore fluid pressure (Bérest 2019; Edirisinghe and Perera 2024; Meng et al. 2024), are prone to trigger damage and failure in the surrounding rocks and associated infrastructures. Morever, the occurrence of discontinuous geological structure and incoherent deformation between surrounding rocks and artifical infrastructures can further exacerbate the risk of damage and failure (Li et al. 2020; Jiang et al. 2021), which has the signtifcicant effects on the overall integrity of storage systems.

In subsurface gas storage systems, sealing integrity issues commonly happen at caprocks and wellbores, resulting in hazards and accidents during long-term operations (Bai et al. 2016; Gudehus et al. 2023; Hartwig et al. 2023). For example, the leakage incident at Aliso Canyon gas storage in the United States was caused by the failure of the SS-25 injection wellbore (Tabibzadeh et al. 2017). Similarly, a survey on new gas wellbores in the United States stated that up to 10% experienced leakage, which was closely associated with damage and failure of caprocks and wellbores (Shukla et al. 2010; Zhang et al. 2023b). The behind reason of wellbore instability is that the range of operating gas pressures is usually designed to ensure salt cavern stability, while the deformation and failure processes of wellbores, critical for sealing integrity, are often overlooked. To ensure stable and long-term performance of subsurface gas storage systems, more attention should be paid to the sealing and contact conditions between the wellbore cement sheath, casing, and surrounding stratum, especially under extreme injection–production cycling.

During long-term operations, the wellbore cement sheath and surrounding rocks are subjected to the extreme temperature (gradient), gas pressure and confining pressure, resulting in complex deformation processes, including expansion and resilience (Davies et al. 2014; Yin et al. 2019). The extremely gas thermo-mechanical enviroment and high-frequency injection–production cycles in long-term operations may induce irreversible and incoherent deformation in the wellbore cement sheath, surrounding rocks, and their contact zones. Some microscopic damage and macroscopic cracks can be often observed in these regions, which can be regareded as gas leakage pathways (De Andrade and Sangesland 2016; Arjomand et al. 2018; He et al. 2022a; Lu et al. 2025), as shown in Fig. 1b. These pathways may result in axial gas leakage along rock strata and the wellbore annulus, thereby increasing the engineering risks associated with wellbore instabilities. Laboratory experiments and field-scale tests have shown that wellbore cement sheaths are more vulnerable than the surrounding rock strata (He et al. 2022b), and they often operate under complex multiphysics environments, such as thermo-mechanical coupling processes (Li et al. 2021). Engineering evidence indicates that once the integrity of the wellbore cement sheath falls below a critical threshold, it becomes challenging to ensure containment stability and long-term sealing performance of underground gas storage (Bérest 2019). Due to in-situ technical constraints and high costs, it is often difficult to synchronously monitor the evolution of multiphysics variables, such as temperature, pressure, gas density, displacement and damage, during operation. Furthermore, the characteristics and underlying failure mechanisms of wellbore cement sheaths under high-frequency injection–production cycles remain insufficiently understood.

The wellbore cement sheath can be regarded as a brittle or quasi-brittle solid material, in which microscopic damage and macroscopic cracks are often observed under complex loading conditions (Arjomand et al. 2018; He et al. 2022c). In recent years, several scholars have investigated the mechanical responses and failure characteristics of wellbore cement sheaths subjected to various loading conditions, such as low-cycle fatigue (Yuan et al. 2013) and high-cycle fatigue (He et al. 2022a, c), through both laboratory experiments (Agofack et al. 2019) and in-situ field-scale tests (Arjomand et al. 2018). For instance, Yuan et al. (2013) conducted low-cycle fatigue experiments on cement to assess the effects of high pressure high temperature (HPHT) on well integrity, aiming to reduce cement failure and improve cement design in HPHT and steam injection wells. Arjomand et al. (2018) performed laboratory experiments to determine the strength properties of Class G cement and subsequently calibrated model parameters based on the experimental data. He et al. (2022c) investigated the fatigue damage of the wellbore cement sheath under different cyclic loading conditions in the laboratory. The experimental data were used to calibrate material parameters in a fatigue damage model, which was applied in numerical simulations of a gas storage salt cavern in Jintan, China. To describe the mechanical behavior of cement sheaths, several constitutive damage models have been proposed, such as elastoplastic damage models (Li et al. 2017), fatigue damage constitutive models (He et al. 2022c), and multiscale damage models (Zhang et al. 2017a). For example, some fatigue damage constitutive models were developed to describe microannuli and microcrack evolution in cement sheaths and rock materials, where parameters can be calibrated through triaxial cyclic loading, NMR imaging, and SEM tests (Liu and Dai 2018, 2021). Similarly, He et al. (2022a) developed an elastoplastic damage constitutive model calibrated by uniaxial/triaxial compression, Brazilian splitting, and cyclic loading tests, and applied it in constitutive modeling and simulations to study cement sheath failure in gas storage salt caverns. However, limitations in synchronized monitoring techniques and the opaque nature of rocks and cements make it difficult to capture the spatiotemporal distribution of multiphysics variables. Consequently, the inherent deformation characteristics and failure mechanisms remain poorly understood.

The rapid developments of computer technology offer an excellent opportunity to simulate the progressive failure process of wellbore cement sheaths. In the past decades, several numerical methods were proposed to simulate the progressive failure processes of cement materials, which can be classified into continuum- and discontinuum-based methods (Zhang et al. 2023a; Zhou and Wang 2021). In the continuum-based framework some numerical methods, such as finite element method (FEM) (He et al. 2022a), extended finite element method (XFEM) (Hong et al. 2024) and peridynamics (PD) (Wang and Wu 2023; Shi et al. 2024), were developed to simulate crack growth paths in wellbore cement sheaths, which is beneficial for assessing the integrity of wellbore cement sheaths and the stability of underground gas storage. However, the classical FE simulations struggle to simulate crack growth paths due to the necessary requirements of remeshing techniques (Trädegård et al. 1998). Moreover, in the discontinuum-based framework, the discrete element method (DEM) was used to simulate wellbore cement sheaths (Gong et al. 2024; Shentu et al. 2024; Lei et al. 2025), with computational parameters calibrated using an empirical trial-and-error strategy. Only clusters of failure regions can be obtained in DEM simulations instead of the accurate trajectories of cracks observed in laboratory and continuum-based numerical simulations, especially in thermo-hydro-mechanical environments. Some other advanced numerical methods, including generalized particle dynamics (Zhou et al. 2015) and the field-enriched FE method (Wang and Zhou 2021), have been developed to simulate the progressive failure of brittle solids, such as rocks, concretes, etc.

In recent years, phase-field method has gained considerable attention as a robust and sophisticated numerical technique for modeling the fracture and failure behavior of solid materials (Francfort and Marigo 1998; Bourdin and Francfort 2000; Wu 2017). Grounded in the principles of energy minimization and variational mechanics (Bourdin et al. 2008; Ambati et al. 2015), this method introduces a scalar phase-field variable to diffuse sharp crack discontinuities over a finite width, thereby reformulating the inherently discontinuous fracture problem into a continuous framework. This approach allows for a unified treatment of crack initiation, propagation, branching, and coalescence, without the need for additional fracture criteria or predefined crack paths (Miehe et al. 2010a). In contrast to traditional discrete crack models, the phase-field method naturally evolves fracture patterns as a result of energetic competition, governed by coupled partial differential equations. The method benefits from a rigorous theoretical foundation and a computationally consistent formulation that aligns well with physical observations (Wu 2018). Furthermore, its numerical implementation is relatively straightforward, and the results have been shown to accurately replicate complex fracture behavior (Miehe et al. 2010b). Owing to its flexibility and predictive capabilities, the phase-field method has demonstrated significant promise in modeling multiscale and multiphsics problems in rock mechanics, including thermo-mechanical coupling (Xia et al. 2023; Nguyen et al. 2023; Kebria et al. 2025), fluid–solid interactions (Yoshioka et al. 2021; Liu et al. 2021), strain localization (Ip and Borja 2022; Wang et al. 2023; Ip and Borja 2023) and frictional fracture (Fei and Choo 2020a, b; Liu et al. 2023; Martogi et al. 2024; Liu and Wang 2025), etc., which are closely related to applications in subsurface gas storage engineering systems. For example, Xu et al. (2024a), Liu and Wang (2025) and Xu et al. (2026) developed phase-field models for capturing frictional damage in pure shear and mixed-mode tension-shear fracture patterns in heterogeneous rocks. Moreover, Xu et al. (2024b) proposed an adaptive strategy in the phase-field framework to simulate 3D internal crack growth in rocks, which has also been extended to explore time-dependent chemical corrosion of sandstone (Xu et al. 2025).

In this work, we propose a computational paradigm for gas thermo-mechanical coupling systems within the well-known phase-field framework. This new numerical tool enhances our understanding of the deformation behavior and failure mechanisms of wellbore cement sheaths in salt cavern gas storage systems. Our proposed computational paradigm has coupled two novel features, including (**i**) a complete theoretical model for gas flow based on thermodynamic principles; and (**ii**) the developed phase-field formulations for this complex gas thermo-mechanical coupling system, associated with a staggered numerical solution strategy. Our approach can not only simulate the evolution of temperature in surrounding rock and gas pressure, but also capture the deformation and failure of the wellbore cement sheath. For both qualitative and quantitative validation, several benchmark examples are presented against analytical solutions, laboratory experimental observations, and field-scale monitoring data. We furthermore carry out a series of numerical simulations to discuss the effects of in-situ geo-stress, critical fracture energy release rate, elastic modulus, and injection–production rate on wellbore integrities and critical operational gas pressures. The relevant deformation characteristics and failure mechanisms are quantitatively analyzed. Importantly, comparisons with field-scale monitoring data confirm the robustness of our computational paradigm and highlight its potential for industrial applications. These numerical simulations help us to propose an optimized injection–production cycling strategy for maximizing subsurface energy storage capability.

The remainder of this paper is organized as follows. Section 2 outlines the thermodynamic governing equations for gas flow. Section 3 introduces the phase-field formulation for the thermo-mechanically coupled system and its numerical solution strategy. Section 4 presents qualitative and quantitative analyses of the spatiotemporal variations of gas thermodynamic parameters under multiphysics conditions, and examines failure evolution and mechanisms of the wellbore cement sheath under thermal–mechanical coupling. Section 5 discusses factors affecting wellbore failure, validates the simulation results against field damage logging data, and proposes the operational optimization measures of storage systems while noting limitations and future work. Section 6 concludes the study. The Appendix provides benchmark problems, a mesh-independence analysis for the phase-field characteristic length, and a nomenclature.

## Governing Equations of Gas Flows in Thermodynamics

The closed-loop injection–production cycle of a salt cavern storage can be regarded as two coupled thermodynamic processes, comprising (i) the flow and heat transfer of gas within the wellbore (i.e., heat transfer between the casing, oil, and cement sheath) and (ii) the thermodynamic state of gas within the cavern, as shown in Fig. 1b. To simulate this thermodynamically coupled gas system, this study developed a model based on the following hypotheses: (i) gas properties are uniform at each depth and vary only with time; (ii) gas flow in the wellbore is considered one-dimensional, steady, and single-phase; (iii) gas within the salt cavern is homogeneous; and (iv) gas leakage is neglected.

Based on our previous theoretical model for gas flow production (Liu et al. 2021), the fluid pressure and temperature of gas can be calculated as $PV = ZnRT_{\text {g}}$, where *P*, *V* and $T_{\text {g}}$ are gas pressure, volume and temperature, respectively; *n* stands for the mole of gas; *R* represents the universal gas constant; and *Z* denotes the gas compressibility, which can be expressed by the Berthelot gas state equation as (Sokovnin et al. 2022)

1 $$\begin{aligned} Z = 1 - \frac{9PT_\textrm{c}}{128T P_\textrm{c}} \left( \frac{6T_\textrm{c}^2}{T_{\text {g}}^2} - 1 \right) , \end{aligned}$$

in which, $P_\textrm{c}$ and $T_\textrm{c}$ are the critical gas pressure and temperature, respectively.

### Gas Flow in Wellbores

To describe the gas pressure in a wellbore, the momemtum equation of a representative volume element (RVE) of gas flow can be expressed as:

2 $$\begin{aligned} \frac{\textrm{d}P}{\textrm{d}z} = \rho g - f \frac{\rho v^2}{4r_o} - \rho v \frac{\textrm{d}v}{\textrm{d}z}, \end{aligned}$$

where z is the wellbore depth, ρ is the gas density, g is the gravitational acceleration, v is the gas flow velocity, and $r_o$ is the inner radius of the metal tubing. The friction coefficient f between the gas and the tubing wall is given by $f = \left[ 1.14 - 2\log _{10}\left( \frac{e}{2r} + \frac{21.25}{\textrm{Re}^{0.9}} \right) \right] ^{-2}$, where e and r are the roughness and radius of the tubing, respectively (Han et al. 2022). The Reynolds number is defined as $\textrm{Re} = \frac{2r_o v \rho }{\mu } = \frac{2\dot{m}}{\mu \pi r_o}$, where $\dot{m}$ is the mass flow rate and μ is the gas viscosity.

According to the first law of thermodynamics, the energy balance equation of gas flow in an RVE can be used to account for the effects of internal energy, external loading work and heat transfer, which can be written as (Han et al. 2022)

3 $$\begin{aligned} q_\textrm{w} = C_\textrm{p}\, dT_{\text {g}} + \frac{9RT_\textrm{c}}{128M P_\textrm{c}} \left( 1 - \frac{18T_\textrm{c}^2}{T_{\text {g}}^2} \right) \textrm{d}P + v\, \textrm{d}v - g\, \textrm{d}z, \end{aligned}$$

where $C_\textrm{p}$ is the gas specific heat at constant pressure, M is the molar mass of the gas, and $q_\textrm{w}$ represents the net heat exchange between the gas flow and the wellbore. The terms on the right-hand side (R.H.S.) of Eq.(3) correspond to: (i) sensible heat effects arising from temperature variations, (ii) real-gas enthalpy corrections due to pressure changes, (iii) kinetic energy variations associated with changes in flow velocity, and (iv) gravitational potential energy differences resulting from elevation changes. The net heat exchange between the gas flow at the representative volume element (RVE) and the wellbore is expressed as

4 $$\begin{aligned} q_\textrm{w} = \frac{2\pi r h_\textrm{w} (T_\textrm{w} - T_{\text {g}})}{\dot{m}}, \end{aligned}$$

where $h_\textrm{w}$ is the heat exchanging coefficient and $T_\textrm{w}$ is the temperature on the inner surface of the wellbore.

### Cavern Thermodynamics

The operational cycle of gas storage typically consists of gas injection, production, and constant-pressure storage stages. Based on the thermodynamic theory for variable-mass systems, Kushnir et al. (2012) proposed a thermodynamic evolution model for compressed air energy storage (CAES). In this study, we adopt their model and express the corresponding mass and energy conservation equations as follows. The governing equation for mass conservation is:

5 $$\begin{aligned} V \frac{\textrm{d}\rho }{\textrm{d}t} = (F_\textrm{i} + F_\textrm{p})\, \dot{m}, \end{aligned}$$

in which V is the volume of the salt cavern, and $F_\textrm{i}$ and $F_\textrm{p}$ are the dimensionless coefficients corresponding to the injection and production stages, respectively.

Moreover, the energy conservation equation of gas in a salt cavern can be expressed as:

6 $$\begin{aligned} & \rho V C_{V0} \frac{\textrm{d}T_{\text {g}}}{\textrm{d}t} = F_\textrm{i} \dot{m}_\textrm{i} C_{P0} (T_\textrm{i} - T_{\text {g}}) + (F_\textrm{i} \dot{m}_\textrm{i} Z_0 R + F_\textrm{p} \dot{m}_\textrm{p} Z_0 R) T_{\text {g}} \nonumber \\ & \quad + (F_\textrm{i} \dot{m}_i + F_\textrm{p} \dot{m}_\textrm{p}) \cdot \frac{\rho R T_{\text {g}0}^2 Z_{T0}'}{\rho _0} + \dot{Q}, \end{aligned}$$

where $C_{V0}$ is the initial specific heat capacity of the gas at constant volume; $T_\textrm{i}$ is the temperature of the injected gas; $\dot{m}_\textrm{i}$ and $\dot{m}_\textrm{p}$ are the mass flow rates of the injected and produced gas, respectively; $C_{P0}$ is the initial specific heat capacity of the gas at constant pressure; $Z_0$ is the compressibility factor at the initial state; $Z_{T0}'$ denotes the temperature derivative of the compressibility factor evaluated at the initial temperature $T_{\text {g}0}$; $\rho _0$ is the gas density at $T_{\text {g}0}$; and $\dot{Q}$ represents the heat transfer rate.

Equation (6) describes the temperature evolution of the gas in a salt cavern. The left-hand side (L.H.S.) represents the rate of change of internal energy due to temperature variation. On the right-hand side (R.H.S.), the first term corresponds to the sensible heat transfer from the injected gas; the second term accounts for the compression or expansion work under compressible gas conditions; the third term provides a correction for the temperature dependence of the compressibility factor; and the last term represents the heat exchange rate with the surrounding rock strata. At the initial temperature $T_0$, the derivative of the compressibility factor with respect to temperature is given by:

7 $$\begin{aligned} Z_{T0}' & = \left( 108 \rho R T_\textrm{c}^3 / (128 P_\textrm{c} T_{\text {g}0}^3)\right) \nonumber \\ & \quad \Big / \left[ 1 + \left( 9 \rho R T_\textrm{c} / (128 P_\textrm{c})\right) \left( 6 T_\textrm{c}^2 / T_{\text {g}0}^2 - 1\right) \right] ^2. \end{aligned}$$

Based on Newton’s law of cooling (Winterton 1999), the heat transfer rate $\dot{Q}$ between the gas flow within cavern and the surrounding rock strata is given by:

8 $$\begin{aligned} \dot{Q} = h_{\text {cv}} A_{\text {cv}} (T_{\text {cv}} - T_{\text {g}}), \end{aligned}$$

where $h_{\text {cv}}$ is the convective heat transfer coefficient, $A_{\text {cv}}$ denotes the effective cavern heat transfer area, and $T_{\text {cv}}$ is the temperature on the cavern surface.

## Thermal-Mechanical Coupled Phase Field Theory

**Fig. 2:**
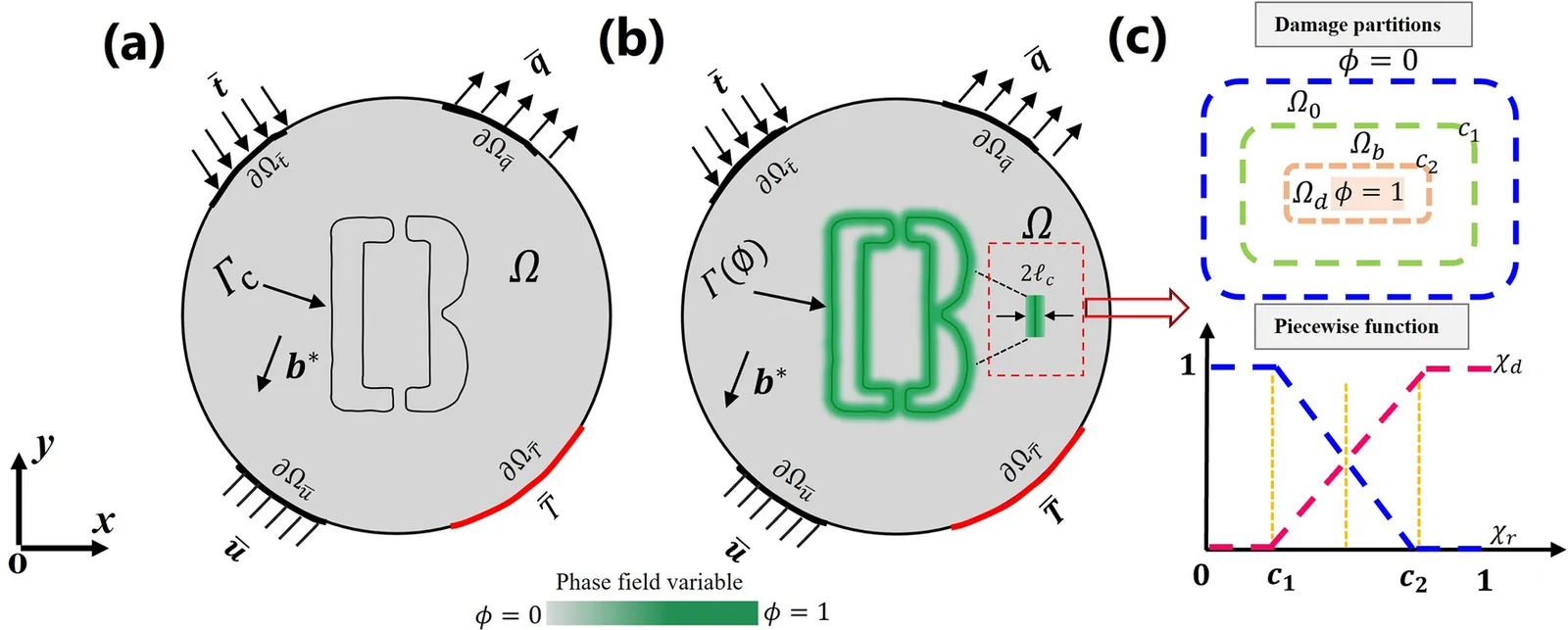
Schematic illustration of a thermo-mechanical coupling system: **a** computational domain with sharp cracks; **b** corresponding diffuse damage zone after regularization in the phase-field framework; and **c** damage partitions represented by interpolated functions

Let us consider arbitrary rock sample occupied the computational domain $\Omega \subseteq \mathbb {R}^{d}$, which is bounded by $\partial \Omega$, as shown in Fig. 2a. In the thermo-mechanical coupling system, the surface boundary $\partial \Omega$ can be classified into Dirichlet boundary (e.g., $\partial \Omega _{\bar{\boldsymbol{u}}}$ and $\partial \Omega _{\bar{T}}$) and Neumann boundary (e.g., $\partial \Omega _{\bar{\boldsymbol{t}}}$ and $\partial \Omega _{\bar{\boldsymbol{q}}}$), which has the following properties: (i) $\partial \Omega = \partial \Omega _{\bar{\boldsymbol{u}}} \cup \partial \Omega _{\bar{\boldsymbol{t}}} = \partial \Omega _{\bar{T}} \cup \partial \Omega _{\bar{\boldsymbol{q}}}$ and (ii) $\partial \Omega _{\bar{\boldsymbol{u}}} \cap \partial \Omega _{\bar{\boldsymbol{t}}} = \emptyset$, $\partial \Omega _{\bar{T}} \cap \partial \Omega _{\bar{\boldsymbol{q}}} = \emptyset$. The computational domain Ω contains crack surfaces $\Gamma _\textrm{c}$, which can be regularized to become a more diffuse damage zone $\Gamma \left( \phi \right)$, as depicted in Fig. 2b.

In this work, we divide regularized diffuse damage region into three zones by the phase-field variable ϕ, including intact zone $\Omega _{0}$, damage transition zone $\Omega _{b}$ and fully fractured zone $\Omega _{d}$, which are used to describe the variation of thermal conductivity across rock progressive failure zones, as shown in Fig. 2c. We can observe that $\phi < c_{1}$ in $\Omega _{0}$; $\phi >c_{2}$ in $\Omega _{d}$; and $c_{1} \le \phi \le c_{2}$ in $\Omega _{b}$, where the interpolated functions can be utilized to compute the effective thermal conductivity.

### Phase-Field Approximation of Crack Surfaces

In the phase-field framework, the sharp crack $\Gamma _\textrm{c}$ is regularized over a more diffuse damage zone $\Gamma \left( \phi \right)$, where $\phi \left( \boldsymbol{x}\right)$ is the phase-field scalar variable. The regularized damage zone $\Gamma \left( \phi \right)$ has the certain thickness related to the internal characteristic length scale $\ell _\textrm{c}$, as shown in Fig. 2b, c. The crack surface density function $\gamma (\phi , \nabla \phi )$ is expressed as

9 $$\begin{aligned} \gamma (\phi , \nabla \phi ) = \frac{\phi ^{2}}{2\ell _\textrm{c}} + \frac{\ell _\textrm{c}}{2} \nabla \phi \cdot \nabla \phi , \end{aligned}$$

where ∇ is the gradient operator.

The crack surface density function $\gamma (\phi , \nabla \phi )$ can be used to build the regularized crack surface area $\Gamma \left( \phi \right)$. Thus, the fracture released energy in the phase-field framework can be written as

10 $$\begin{aligned} \int _{\Gamma } \mathcal {G}_\textrm{c} \, \textrm{d}S = \int _{\Omega } \mathcal {G}_\textrm{c} \left[ \frac{\phi ^{2}}{2 \ell _\textrm{c}} + \frac{\ell _\textrm{c}}{2} \nabla \phi \cdot \nabla \phi \right] \, \textrm{d}\Omega , \end{aligned}$$

in which, $\mathcal {G}_\textrm{c}$ stands for the critical fracture energy release rate of cements, which can be measured by laboratory experiments, such as three-point bending and semi-circular bending tests.

### Energy Decomposition Strategy

In the phase-field framework, the total potential energy function of this system can be expressed as the sum of stored elastic strain energy $\Psi _{e}$, fracture surface released energy $\Psi _{f}$ and external loading work $W_{\text {ext}}$.

11 $$\begin{aligned} & \Psi (\boldsymbol{u}, \Gamma _\textrm{c}) = \underbrace{\int _{\Omega \setminus \Gamma _\textrm{c}} \psi _{e}(\boldsymbol{\varepsilon }) \, \textrm{d}\Omega }_{\begin{array}{c} =\Psi _{e} \end{array}} + \underbrace{\int _{\Gamma _\textrm{c}} \mathcal {G}_\textrm{c} \, \textrm{d}S}_{\begin{array}{c} = \Psi _{f} \end{array}} \nonumber \\ & \quad - \underbrace{\int _{\Omega } \boldsymbol{b}^{*} \cdot \boldsymbol{u} \, d\Omega - \int _{\partial \Omega } \bar{\boldsymbol{t}} \cdot \boldsymbol{u} \, \textrm{d}S}_{=W_{\text {ext}}}, \end{aligned}$$

where $\boldsymbol{b}^{*}$ is the body force density, $\bar{\boldsymbol{t}}$ denotes the prescribed traction along $\partial \Omega _{\bar{\boldsymbol{t}}}$, and $\psi _{e}$ is the stored strain energy density function, expressed in terms of the strain tensor $\boldsymbol{\varepsilon } = \frac{1}{2} \left( \nabla \boldsymbol{u} + (\nabla \boldsymbol{u})^{T} \right)$, with $\boldsymbol{u}$ being the displacement field. This function is defined for an isotropic elastic solid as:

12 $$\begin{aligned} \psi _e(\boldsymbol{\varepsilon }) = \frac{1}{2} \lambda \left( \textrm{tr}(\boldsymbol{\varepsilon }) \right) ^2 + \mu \, \boldsymbol{\varepsilon } : \boldsymbol{\varepsilon }, \end{aligned}$$

in which $\lambda = E \nu / \left[ \left( 1+\nu \right) \left( 1-2\nu \right) \right]$ and $\mu = \frac{E}{2(1 + \nu )}$ are Lamé parameters with Young’s modulus *E* and Poisson’s ratio ν.

In the salt cavern gas storage system, the effect of injection–production cycles during operation on the deformation and failure characteristics of wellbore cement sheaths is closely related to temperature and in-situ geostress conditions. In this thermo-mechanical coupling system, we consider the temperature differences and initial geostress conditions, resulting in the new total potential energy function $\mathcal {E}\left( \boldsymbol{u}, \Delta T, \boldsymbol{\sigma }_{0}, \phi \right)$.

13 $$\begin{aligned} \begin{aligned} \mathcal {E}(\boldsymbol{u}, \Delta T, \boldsymbol{\sigma }_{0}, \phi ) :=&\underbrace{\int _{\Omega } \psi _{e}(\boldsymbol{\varepsilon }(\boldsymbol{u}), \Delta T, \phi ) \, \textrm{d}\Omega }_{= \Psi _{e}\left( \boldsymbol{u}, \Delta T, \phi \right) } + \underbrace{\int _{\Omega } \boldsymbol{\sigma }_0 : \boldsymbol{\varepsilon } \, \textrm{d}\Omega }_{= \Psi _{0}\left( \boldsymbol{\sigma }_{0}, \boldsymbol{u}\right) }\\&+ \underbrace{\int _{\Omega } \mathcal {G}_\textrm{c} \gamma \left( \phi , \nabla \phi \right) , d\Omega }_{\Psi _{f}\left( \phi , \nabla \phi \right) } \\&- \underbrace{\int _{\Omega } \boldsymbol{b}^{*} \cdot \boldsymbol{u} \textrm{d}\Omega - \int _{\partial \Omega } \bar{\boldsymbol{t}} \cdot \boldsymbol{u} \, \textrm{d}\partial \Omega }_{= \Psi _{\text {ext}}\left( \boldsymbol{u}\right) } \end{aligned}, \end{aligned}$$

where $\boldsymbol{\varepsilon }$ is the total strain tensor that can be decomposed into $\boldsymbol{\varepsilon }_{e} = \boldsymbol{\varepsilon } - \boldsymbol{\varepsilon }_{T}$. $\boldsymbol{\varepsilon }_{e}$ is the strain tensor in elasticity and $\boldsymbol{\varepsilon }_{T}$ is thermal strain tensor due to temperature differences, which can be expressed as $\boldsymbol{\varepsilon }_T(\textbf{u}) = \beta \Delta T \boldsymbol{I}$ with the temperature difference of ΔT and the thermal expansion coefficient of β.

Based on the strain spectrum decomposition strategy (Miehe et al. 2010a, b), we decompose the elastic strain tensor in to the tensile and compressive components as follows,

14 $$\begin{aligned} \left\{ \begin{aligned} \boldsymbol{\varepsilon }_{e}^{+}&= \sum \nolimits _{a=1}^{d} \langle \varepsilon _a^e \rangle _+ \, \boldsymbol{n}_a \otimes \boldsymbol{n}_a \\ \boldsymbol{\varepsilon }_e^-&= \sum \nolimits _{a=1}^{d} \langle \varepsilon _a^e \rangle _- \, \textbf{n}_a \otimes \textbf{n}_a \end{aligned} \right. . \end{aligned}$$

in which, $\boldsymbol{\varepsilon }_{\pm }^{e}$ denote the tensile and compressive components of the elastic strain tensor, respectively; $\varepsilon _{a}^{e}$ and $\boldsymbol{n}_{a}$ represent the principal strain and its direction. The Macaulay brackets are defined as $\langle \cdot \rangle _{+} = (\cdot + |\cdot |)/2$ and $\langle \cdot \rangle _{-} = (\cdot - |\cdot |)/2$. The stored elastic strain energy density $\psi _{e}$ in cement materials can also be decomposed into a tensile part $\psi _{e}^{+}$ and a compressive part $\psi _{e}^{-}$, induced by $\boldsymbol{\varepsilon }_{+}^{e}$ and $\boldsymbol{\varepsilon }_{-}^{e}$, respectively.

15 $$\begin{aligned} \left\{ \begin{aligned} \psi _{e}^{+}(\boldsymbol{\varepsilon }, \Delta T)&= \frac{\lambda }{2} \langle \textrm{Tr}(\boldsymbol{\varepsilon }-\beta \Delta T \boldsymbol{I}) \rangle _+^2 + \mu \, \textrm{Tr}\left\{ (\boldsymbol{\varepsilon }-\beta \Delta T \boldsymbol{I})_{-}^{2}\right\} \\ \psi _e^{-}(\boldsymbol{\varepsilon }, \Delta T)&= \frac{\lambda }{2} \langle \textrm{Tr}(\boldsymbol{\varepsilon }-\beta \Delta T \boldsymbol{I}) \rangle _-^2 + \mu \, \textrm{Tr}\left\{ (\boldsymbol{\varepsilon }-\beta \Delta T \boldsymbol{I})_{-}^{2}\right\} \end{aligned} \right. . \end{aligned}$$

In this work, the damage initiation and progressive failure process in wellbore cement sheaths are assumed to be caused by the thermo-mechanical coupling tensile and mixed-mode shear stress, which is driven by the tensile part $\psi _{e}^{+}(\boldsymbol{\varepsilon }, \Delta T)$. While, the compressive part $\psi _{e}^{-}(\boldsymbol{\varepsilon }, \Delta T)$ does not contribute to the damage and failure in wellbore cement sheaths. Now, we recast the stored elastic strain energy function in a thermo-mechanical coupling system as

16 $$\begin{aligned} \int _{\Omega } \psi _{e} \left( \boldsymbol{u}, \Delta T, \phi \right) d\Omega = \int _{\Omega } \left[ \text {g}_{1}\left( \phi \right) \psi _{e}^{+} \left( \boldsymbol{u}, \Delta T\right) + \psi _{e}^{-} \left( \boldsymbol{u}, \Delta T\right) \right] d\Omega , \end{aligned}$$

where $\text {g}_{1} \left( \phi \right)$ is the degradation function to describe the residual stored elastic strain energy in the computational domain, which can be written as

17 $$\begin{aligned} \text {g}_{1} \left( \phi \right) = \left[ \left( 1-\phi \right) ^{2} + \kappa \right] , \end{aligned}$$

in which, κ is a positive constant to overcome the numerical instability.

For wellbore cement sheaths in salt cavern gas storage, the initial damage and its degradation effect due to in-situ geostress are also taken into consideration. Therefore, the second term on R.H.S of Eq. (13) can be approximated as

18 $$\begin{aligned} \Psi _{0}\left( \boldsymbol{\sigma }_{0}, \boldsymbol{u}\right) = \int _{\Omega } \boldsymbol{\sigma }_{0} : \boldsymbol{\varepsilon }\left( \boldsymbol{u}\right) \, \textrm{d}\Omega \simeq \int _{\Omega } \text {g}_{1} \left( \phi \right) \, \boldsymbol{\sigma }_0 : \boldsymbol{\varepsilon }\left( \boldsymbol{u}\right) \, \textrm{d}\Omega . \end{aligned}$$

### Heat Transfer Model

To build the gas thermo-mechanical coupling computing paradigm for numerical analysis of salt cavern gas storage subjected to operational cycles, we also develop a heat transfer model for describing the temperature variation in wellbore cement sheaths and cavern surrounding rocks as follows,

19 $$\begin{aligned} \left\{ \begin{array}{ll} \rho _{sw} c_{psw} \dot{T}_{sw} + \nabla \cdot \boldsymbol{q} = 0 & \text {in} \quad \Omega _\textrm{w} \\ \boldsymbol{q} \cdot \boldsymbol{n} = q^* & \text {on} \quad \partial \Omega _\textrm{w} \\ \end{array} \right. , \end{aligned}$$

20 $$\begin{aligned} \left\{ \begin{array}{ll} \rho _{sc} c_{psc} \dot{T}_{sc} + \nabla \cdot \boldsymbol{Q} = 0 & \text {in} \quad \Omega _{ca} \\ \boldsymbol{Q} \cdot \boldsymbol{n} = Q^* & \text {on} \quad \partial \Omega _{ca} \end{array} \right. , \end{aligned}$$

where $\Omega _{w}$ and $\Omega _{ca}$ are the computational regions for wellbore cement sheaths and cavern surrounding rocks, respectively; $\rho _{sw} c_{psw}$ and $\rho _{sc} c_{psc}$ represent the volumetric heat capacities for these two regions, consisting of densities $\rho _{\left( \bullet \right) }$ and specific heat capacities $c_{\left( \bullet \right) }$ of materials with $\left( \bullet \right) = sw$ for wellbore cement sheaths and $\left( \bullet \right) = sc$ for cavern surrounding rocks, respectively. Similarly, $\dot{T}_{sw}$ and $\dot{T}_{sc}$ stand for rates of temperature variation in $\Omega _{w}$ and $\Omega _{ca}$, respectively.

According to the classical Fourier’s law, the heat fluxes $\boldsymbol{q}$ and $\boldsymbol{Q}$ on surfaces of wellbore cement sheaths and cavern surrounding rocks can be written as

21 $$\begin{aligned} \left\{ \begin{aligned} \boldsymbol{q}&= -k_{sw} \cdot \nabla T_{sw} \\ \boldsymbol{Q}&= -k_{sc} \cdot \nabla T_{sc} \end{aligned} \right. , \end{aligned}$$

in which, $k_{sw}$ and $k_{sc}$ are the thermal conductivity of wellbore cement sheath and cavern surrounding rock, respectively.

Taking the damage and micro-cracks in the wellbore cement sheath into consideration, in analogy to fluid conductivity in hydraulic fracture (Zhou et al. 2020; Liu et al. 2025), we define an effective thermal conductivity $k_{\text {eff}}$ to account for the crack-induced thermal resistance effect as follows,

22 $$\begin{aligned} k_{\text {eff}} = k_{r} \chi _{r} + k_{d} \chi _{d}, \end{aligned}$$

where $\chi _{r}$ and $\chi _{d}$ are interpolation functions to describe the degradation of thermal conductivity from the intact region, i.e., $k_{r}$, to the fully cracked region, i.e., $k_{d}$, as illustrated in Fig. 2c. Two interpolation functions can be written as

23 $$\begin{aligned} \begin{aligned} \chi _r(\cdot , \phi )&= {\left\{ \begin{array}{ll} 1, & \phi \le c_1 \\ \frac{c_2 - \phi }{c_2 - c_1}, & c_1< \phi< c_2 \\ 0, & \phi \ge c_2 \end{array}\right. } \\ \qquad \chi _{d}(\cdot , \phi )&= {\left\{ \begin{array}{ll} 0, & \phi \le c_1 \\ \frac{\phi - c_1}{c_2 - c_1}, & c_1< \phi < c_2 \\ 1, & \phi \ge c_2 \end{array}\right. } \end{aligned}. \end{aligned}$$

Figure 2c and Eq. (23) demonstrate that heat transferring behavior in both wellbore cement sheaths and cavern surrounding rocks can be categorized into three regions by the phase-field variable ϕ, including a intact zone ($\phi < c_{1}$), a fully cracked zone ($\phi >c_{2}$) and a transition zone ($c_{1}\le \phi \le c_{2}$). In this work, we only focus on the damage and failure characteristics of wellbore cement sheaths. It is noted that the width of a transition zone is dominated by the internal characteristic length $\ell _\textrm{c}$ within the phase-field framework, which is a nonlocal parameter of materials, similar to *horizon* size in peridynamics (Wang et al. 2018a, 2019a). This numerical treatment allows for continuous variation of the thermal conductivity across a specific computational domain of salt cavern gas storage.

In the realistic salt cavern gas storage, heat transfer happens on (i) the surrounding wellbore surface $\mathcal {A}_{w}$, which is a local, narrow and tabular contact interface between the wellbore cement sheath and the surrounding host salt/rock formation, and (ii) the cavern surface $\mathcal {A}_\textrm{c}$ that is a large-scale, irregular, and internal boundary of the underground cavern. It is noted that the surrounding wellbore surfaces and cavern surfaces are connected at the wellbore–cavern junction and are coupled via mechanical and thermal interactions. Thus, the Robin boundary conditions on these two surfaces are given by

24 $$\begin{aligned} \left\{ \begin{aligned} - k_{\text {eff}} \cdot \nabla T_{sw}&= h_{sw}(T_{sw} - T_{f}), \quad & \text {on} \quad \mathcal {A}_{w} \times \left[ 0, T\right] \\ - k_{sc} \cdot \nabla T_{sc}&= h_{sc}(T_{sc} - T_{f}), \quad & \text {on} \quad \mathcal {A}_\textrm{c} \times \left[ 0, T\right] \end{aligned} \right. , \end{aligned}$$

where $h_{sw}$ is the convective heat transfer coefficient on the surrounding wellbore surfaces, quantifying the exchange of heat between injected/stored fluid inside the wellbore and the relevant cement sheath; $h_{sc}$ denotes the convective heat-transfer coefficient on the cavern surfaces, quantifying exchanges of heat between the salt cavern walls and stored fluids in cavern storage; and $T_{f}$ represent temperature of the injected/stored fluid, as a reference temperature in the heat transfer model. While, for the Dirichlet boundary in this heat transfer model, we take $T_{sw} = T_{w0}$ on $\mathcal {A}_{w}$ and $T_{sc} = T_{c0}$ on $\mathcal {A}_\textrm{c}$.

### Formulations for Thermo-mechanical Coupling Processes

In the thermo-mechanical coupling salt cavern gas storage system, considering the strain energy decomposition strategy, contribution of in-situ stress and potential initial damage state in wellbore cement sheaths, we can reformulate the new potential energy function in Eq. (13) as,

25 $$\begin{aligned} \begin{aligned}\mathcal {E}(\boldsymbol{u}, \Delta T, \boldsymbol{\sigma }_{0}, \phi ) & := \underbrace{\int _{\Omega } \left[ \text {g}_{1}\left( \phi \right) \psi _{e}^{+}\left( \boldsymbol{u}, \Delta T\right) + \psi _{e}^{-} \left( \boldsymbol{u}, \Delta T\right) \right] \textrm{d}\Omega }_{\text {TM coupling stored energy } \Psi _{e}} \\& \quad + \underbrace{\int _{\Omega } \text {g}_{1}\left( \phi \right) \boldsymbol{\sigma }_{0} : \boldsymbol{\varepsilon }\left( \boldsymbol{u}\right) \textrm{d}\Omega }_{\text {In-situ stress term } \Psi _0} \\& \quad + \underbrace{\int _{\Omega } \mathcal {G}_\textrm{c} \left[ \frac{\phi ^{2}}{2\ell _\textrm{c}} + \frac{\ell _\textrm{c}}{2} \nabla \phi \cdot \nabla \phi \right] \textrm{d}\Omega }_{\text {Fracture released energy } \Psi _f} \\& \quad - \underbrace{\left( \int _{\Omega } \boldsymbol{b}^{*} \cdot \boldsymbol{u} d\Omega + \int _{\partial \Omega } \bar{\boldsymbol{t}}\cdot \boldsymbol{u} \textrm{d}\Omega \right) }_{\text {External loading work } \Psi _{\text {ext}}} \end{aligned} . \end{aligned}$$

The variational phase-field framework for fracture aims to find the admissible displacement field $\boldsymbol{u}$ and phase-field variable ϕ, which can be regarded as a minimization optimization problem (Bourdin and Francfort 2000; Bourdin et al. 2008). Based on the minimization potential energy principle in continuum mechanics, we can find the stationary point using the variational principle as follows,

26 $$\begin{aligned} \delta \mathcal {E}(\boldsymbol{u}, \Delta T, \phi , \delta \boldsymbol{u}, \delta \phi ) = 0 \end{aligned}$$

In contrast to the variational consistent formulation, the variationally inconsistent hybrid formulation was proposed to solve both weakly coupled minimization problems simultaneously, referring to Wu (2018).

Now, the resulting constitutive relations, i.e., Cauchy stress tensor $\boldsymbol{\sigma }_{e}$, reads (Liu et al. 2022, 2023; Liu and Wang 2025)

27 $$\begin{aligned} \boldsymbol{\sigma }_{e} = \mathbb {D} : \boldsymbol{\varepsilon }_{e} = \text {g}_{1}\left( \phi \right) \frac{\partial \psi _{e}^{+}(\varepsilon _{ij}, \Delta T)}{\partial \varepsilon _{ij}} + \frac{\partial \psi _{e}^{-}(\varepsilon _{ij}, \Delta T)}{\partial \varepsilon _{ij}}, \end{aligned}$$

which can be explicitly stated as

28 $$\begin{aligned} \begin{aligned} \boldsymbol{\sigma }_{e} =&\left[ \left( 1-\phi \right) ^{2}+\kappa \right] \left[ \lambda \, \langle \operatorname {Tr} \left( \boldsymbol{\varepsilon }-\beta \Delta T \boldsymbol{I} \right) \rangle _{+} \boldsymbol{I} + 2\mu \, \boldsymbol{\varepsilon }_{+} \right] \\&+ \lambda \, \langle \operatorname {Tr} \left( \boldsymbol{\varepsilon }-\beta \Delta T \boldsymbol{I} \right) \rangle _{-} \boldsymbol{I} + 2\mu \, \boldsymbol{\varepsilon }_{-} \end{aligned}, \end{aligned}$$

where $\mathbb {D}$ is the fourth-order elastic stiffness tensor expressed as (Zhou et al. 2018; Xia et al. 2023)

29 $$\begin{aligned} \mathbb {D}&= \frac{\partial \boldsymbol{\sigma }}{\partial \boldsymbol{\varepsilon }} = \lambda \left\{ \left[ \left( 1-\phi \right) ^{2} + \kappa \right] \mathcal {H}_{\varepsilon } \left( \textrm{Tr}(\boldsymbol{\varepsilon })\right) + \mathcal {H}_{\varepsilon }\left( -\textrm{tr}\left( \boldsymbol{\varepsilon }\right) \right) \right\} \boldsymbol{I} \otimes \boldsymbol{I} \end{aligned}$$

30 $$\begin{aligned}&\quad + 2\mu \left\{ \left[ \left( 1-\phi \right) ^{2} + \kappa \right] \frac{\partial \boldsymbol{\varepsilon }_+}{\partial \boldsymbol{\varepsilon }} + \frac{\partial \boldsymbol{\varepsilon }_-}{\partial \boldsymbol{\varepsilon }} \right\} , \end{aligned}$$

in which, $\mathcal {H}_{\varepsilon }\left( x\right)$ is the Heaviside function defined as $\mathcal {H}_{\varepsilon }\left( x\right) = 1.0$ for x>0 and $\mathcal {H}_{\varepsilon }\left( x\right) = 0.0$ for x≤0. The fourth-order constitutive tensor $\mathbb {D}_{ijkl}$ is decomposed into two parts,

31 $$\begin{aligned} \mathbb {D}_{ijkl} = \bar{\mathbb {D}}_{ijkl} + \tilde{\mathbb {D}}_{ijkl} \end{aligned}$$

where each component can be expressed as (Zhou et al. 2018),

32 $$\begin{aligned} \left\{ \begin{aligned} \bar{\mathbb {D}}_{ijkl}&= \lambda \left\{ \left[ \left( 1-\phi \right) ^{2} + \kappa \right] \mathcal {H}_{\varepsilon } \left( \textrm{Tr}(\boldsymbol{\varepsilon })\right) + \mathcal {H}_{\varepsilon }\left( -\textrm{tr}\left( \boldsymbol{\varepsilon }\right) \right) \right\} \delta _{ij} \delta _{kl} \\ \tilde{\mathbb {D}}_{ijkl}&= 2\mu \left\{ \left[ \left( 1-\phi \right) ^{2} + \kappa \right] \mathbb {P}_{ijkl}^{+} + \mathbb {P}_{ijkl}^{-} \right\} \end{aligned} \right. , \end{aligned}$$

where

33 $$\begin{aligned} \mathbb {P}_{ijkl}^{\pm } = \mathbb {P}_{1ijkl}^{\pm } + \mathbb {P}_{2ijkl}^{\pm } \end{aligned}$$

with

34 $$\begin{aligned} \left\{ \begin{aligned} \mathbb {P}_{1ijkl}^{\pm }&= \sum _{a=1}^{3} \sum _{b=1}^{3} \mathcal {H}_{\varepsilon }(\pm \varepsilon _a) \delta _{ab} \, n_{ai} n_{aj} n_{bk} n_{bl} \\ \mathbb {P}_{2ijkl}^{\pm }&= \sum _{a=1}^{3} \sum _{\begin{array}{c} b=1 \\ b \ne a \end{array}}^{3} \frac{1}{2} \frac{ \langle \varepsilon _a \rangle ^{\pm } - \langle \varepsilon _b \rangle ^{\pm } }{ \varepsilon _a - \varepsilon _b } n_{ai} n_{bj} ( n_{ak} n_{bl} + n_{bk} n_{al} ) \end{aligned} \right. , \end{aligned}$$

in which $\varepsilon _{a}$ is the principal strain components; $n_{ai}$ represents the *i*-th component of the principal strain direction vector $\boldsymbol{n}_{a}$. To avoid the singularity in numerical simulations, we follow the idea by Zhou et al. (2018) to incorporate a perturbation term for the principal strains, which reads,

35 $$\begin{aligned} \left\{ \begin{aligned} \varepsilon _1&= \varepsilon _1 (1 + \delta ) \quad&\text {if } \varepsilon _1 = \varepsilon _2 \\ \varepsilon _3&= \varepsilon _3 (1 - \delta ) \quad&\text {if } \varepsilon _2 = \varepsilon _3 \end{aligned} \right. , \end{aligned}$$

where $\delta =1.0\times 10^{-9}$ is the value of artificial perturbation, leading to the unchanged second principal strain.

Based on the standard variational principles, the Euler–Lagrange equations coupled with the irreversible conditions of phase-field evolution, the strong form of governing equations for describing the thermo-mechanical coupling process can be written as

36a $$\begin{aligned}&\nabla \cdot \boldsymbol{\sigma } \left( \boldsymbol{u}, \Delta T, \boldsymbol{\sigma }_{0}, \phi \right) + \boldsymbol{b} = \boldsymbol{0}, \end{aligned}$$

36b $$\begin{aligned}&\left[ \frac{2\ell _\textrm{c}\left( 1-\phi \right) \mathcal {H}}{\mathcal {G}_\textrm{c}} + 1\right] \phi - \ell _\textrm{c}^{2} \nabla ^{2}\phi = 0, \end{aligned}$$

36c $$\begin{aligned}&\rho c \dot{T} + \nabla \cdot \boldsymbol{q} = 0 . \end{aligned}$$.

To ensure robustness and physical admissibility in phase-field modeling, the irreversibility condition must be satisfied, namely $\gamma (x,s) \in \gamma (x,t)$ for all s≤t. A common employed strategy to enforce this requirement is the introduction of a history field $\mathcal {H}\left( x,t\right)$, which stores the maximum tensile elastic energy density attained during the loading history. Specifically, $\mathcal {H}\left( x,t\right)$ denotes the maximum positive strain energy accumulated at position *x* up to time *t*, and is defined as:

37 $$\begin{aligned} \mathcal {H}\left( x,t\right) = \max _{s \in [0,t]} \left\{ \psi _e^+(x,s) + \boldsymbol{\sigma }_0 : \boldsymbol{\varepsilon }(x,s) \right\} , \quad \text {in } \Omega \times (0,T]. \end{aligned}$$

Moreover, the boundary conditions (i.e., Dirichlet and Neumann types) and initial conditions for the governing equations of displacement field, temperature, and phase-field variable in the thermo-mechanical coupling system can be specified as follows,

38a $$\begin{aligned}&\boldsymbol{\sigma } \cdot \boldsymbol{n} = \bar{\boldsymbol{t}} \qquad & \text {on} \quad \partial \Omega _{t} \times \left[ 0, t\right] , \end{aligned}$$

38b $$\begin{aligned}&\boldsymbol{u}\left( \boldsymbol{x}, t\right) = \bar{\boldsymbol{u}} \qquad & \text {on} \quad \partial \Omega _{\boldsymbol{u}} \times \left[ 0, t\right] , \end{aligned}$$

38c $$\begin{aligned}&T \left( \boldsymbol{x}, t\right) = \bar{T} & \text {on} \quad \partial \Omega _{\bar{T}} \times \left[ 0, t\right] , \end{aligned}$$

38d $$\begin{aligned}&\nabla \phi \cdot \boldsymbol{n} = 0 \qquad & \text {on} \quad \partial \Omega \times \left[ 0, t\right] , \end{aligned}$$

38e $$\begin{aligned}&\boldsymbol{q} \cdot \boldsymbol{n} = q^{*} & \text {on} \quad \partial \Omega _{\boldsymbol{q}} \times \left[ 0, t\right] , \end{aligned}$$

38f $$\begin{aligned}&\phi \left( \boldsymbol{x}, t = 0\right) = \phi _{0} \qquad & \text {in} \quad \Omega , \end{aligned}$$

38g $$\begin{aligned}&\boldsymbol{u}\left( \boldsymbol{x}, t=0\right) = \boldsymbol{u}_{0} \qquad & \text {in} \quad \Omega . \end{aligned}$$

### Numerical Implementation and Solution Strategy

**Fig. 3:**
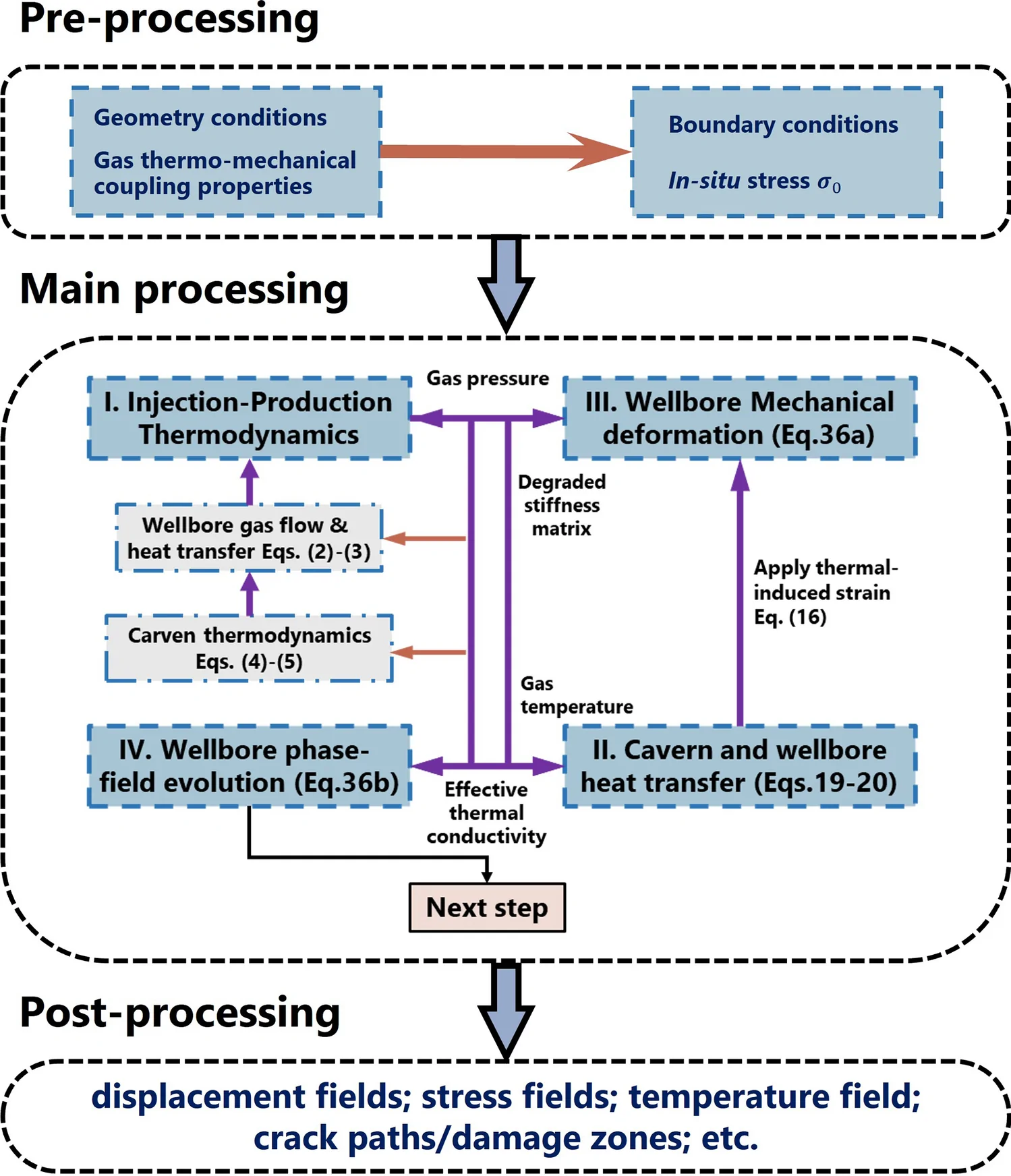
Computing flowchart of the developed gas thermo-mechanical phase-field model

The developed gas themo-mechanical phase-field model is implemented within a finite element (FE) framework. The governing equations of heat transfer, linear momentum balance, and phase-field fracture evolution are fully integrated with the associated numerical solution strategy (Liu et al. 2022, 2023; Liu and Wang 2025), as illustrated schematically in Fig. 3.

The simulation starts with a thermodynamic ODE model of the gas injection–production cycle, discretized by finite differences to obtain cavern pressure and temperature. These serve as boundary conditions for the wellbore, where themo-mechanical integrity of cement sheaths is assessed. Coupling is achieved via the phase-field variable, which governs stiffness degradation. Gas pressure drives deformation, temperature induces heat transfer and thermal strains, and both feed into the phase-field evolution that updates conductivity and stiffness.

To solve the coupled governing equations, a staggered scheme solves the coupled governing equations, with thermodynamic, heat transfer, mechanical, and phase-field subproblems updated sequentially within each time step. Strong coupling is enforced through iterative updates, while nonlinearities from multiphysics and constitutive effects are handled by consistent stiffness and load updates. Time integration uses the generalized-α method for stability and damping, and the nonlinear algebraic system is solved by Newton–Raphson with Anderson acceleration to improve convergence.

This computational framework ensures stable and accurate integration of gas thermodynamics with themo-mechanical and fracture processes, enabling reliable prediction of wellbore integrity under complex storage operations. Furthermore, several benchmarks are presented for numerical validation in the Appendix.

## Numerical Simulations and Results

**Fig. 4:**
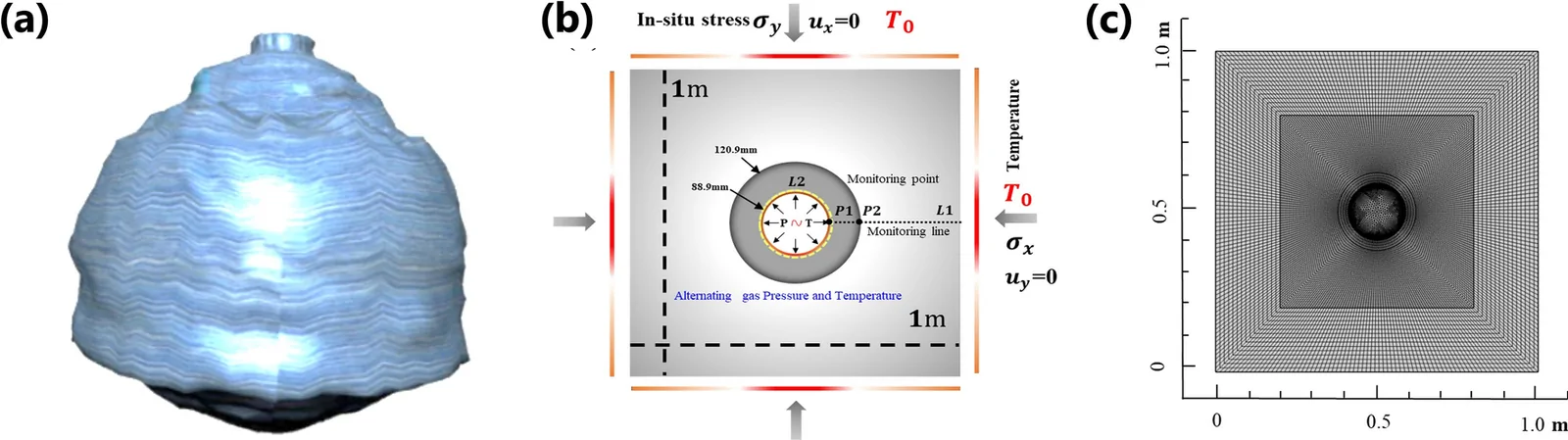
**a** Geometry of the T-5 salt cavern in Jintan determined from sonar survey (Liu et al. 2021). **b** Geometric and boundary conditions of a numerical sample with monitoring lines (*L*1, *L*2) and points (*P*1, *P*2). **c** Finite element mesh after convergence analysis

In this section, we apply our gas thermo-mechanical phase-field approach to simulate the deformation and progressive failure of wellbore cement sheaths in a real-world case. Jintan salt cavern underground gas storage (UGS) is the first and representative UGS in China, where the working gas capacity of about 1.7 × 10$^{9}$ m$^{3}$ (Wang et al. 2019c). In this case, we focus on the numerical analysis on wellbore integrity in Jintan T5-2 UGS during its operation stage (Liu et al. 2021), as shown in Fig. 4a. In our simulations, we first investigate the spatiotemporal evolution of thermodynamic parameters within the gas storage. We then examine the kinetics of damage and cracking in the cement sheath under coupled themo-mechanical loading. Finally, we clarify the mechanical mechanisms governing cement-sheath damage and cracking. All analyses are performed both qualitatively and quantitatively.

### Geometric and Boundary Conditions

In the numerical analysis, we adopt the plane-strain hypothesis to simulate the deformation and progressive failure of wellbore cement sheaths. The numerical model is defined as a square region of interest with a side length of 1 m, containing one wellbore at the center, as shown in Fig. 4b. It shows that the surrounding rocks are represented by the light-colored areas, whereas the dark gray regions denote the cement sheath. The cement sheath has an inner diameter of 89.90 mm and an outer diameter of 120.90 mm, and it is in direct contact with the surrounding stratum.

In the deep salt caverns, the isotropic in-situ stress state is seldom observed due to the presence of microcracks, heterogeneity and multiphysics coupling effects, as demonstrated by field tests on hydraulic fracturing technology in petroleum/gas engineering applications (Jiang et al. 2025). Thus, we consider the differences between horizontal and vertical in-situ stresses applied on boundaries. In steadily deposited rock salt formations, vertical in-situ stress usually plays the dominated role, whereas the maximum horizontal one is approximately 0.4–1.0 times the vertical one, which serves as a basis for the numerical model boundaries. Moreover, the initial formation temperature is set to $T_{0}$ and maintained as a constant boundary condition during UGS operation, as shown in Fig. 4b. Gas pressure and the evolving temperature are applied on the inner boundary surfaces of a wellbore cement sheath as Neumann and Dirichlet boundary conditions, respectively. We summarize the geometric parameters and initial values of numerical samples in Table 1. To capture the thermo-mechanical coupling responses and the spatiotemporal evolution of cracks, two monitoring lines (L1 and L2) and two monitoring points (P1 and P2) are introduced into the numerical model.

**Table 1:** Geometric and boundary conditions in the numerical sample (Liu et al. 2021)

Parameter	Unit	Value	Parameter	Unit	Value
Cavern volume	m^3^	$1.883 \times 10^{5}$	Tubing roughness	m	$1.5 \times 10^{-5}$
Cavern surface area	m^2^	$1.197 \times 10^{4}$	Initial gas pressure	Pa	$9.0 \times 10^{6}$
Wellbore length	m	1016.0	Initial gas temperature	K	303.15
Tubing diameter	mm	177.8	Injected gas temperature	K	308.15

The material parameters as well as the thermodynamic properties of natural gas are summarized in Table 2, which are all temperature-independent. It is worth noted that the reported investigations mainly focused on the cavern stability and the relevant creep shrinkage behavior, while the wellbore integrity is often overlooked. Compared with the high in-situ stress conditions at cavern depths, the wellbore, particularly in the near wellhead section, is subjected to the low in-situ stress and thus performs greater susceptibility to tensile damage during operation. In our numerical simulations, the horizontal in-situ stress on one side is set as 7 MPa, whereas lateral pressure coefficient on the other side is employed as $K_\textrm{c} = 1.3$ to achieve the realistic situation. Moreover, the working pressure, i.e., gas pressure, on internal surfaces of wellbores, ranges from 9.0 to 19.0 MPa to investigate the thermo-mechanical responses and progressive failure characteristics of wellbore cement sheaths, according to the recorded value of 7–17 MPa (He et al. 2022c; Liu et al. 2021).

Each operation cycle spans 24 days, consisting of four sequential stages: gas injection (8 days), high-pressure storage (4 days), production (8 days), and low-pressure storage (4 days). The average gas injection and production rate is approximately 1.2 MPa/day. This loading scheme is designed for the preliminary investigation on deformation and failure characteristics of cement sheaths at the depth of about 300 m, where the extreme gas thermo-mechanical coupling is considered for the intensive injection–production cycles.

Based on reported peak strengths of the surrounding rock and cement sheath (He et al. 2022a, c), the critical fracture energy release rates are estimated to be about 120.0 N/m and 100.0 N/m, respectively. These values are obtained via the inverse calculations using Griffith’s criterion and Irwin’s energy-based fracture theory, which shows good agreement with previous studies (Xia et al. 2023). In our phase-field simulations, we also adopt the characteristic length as $\ell _\textrm{c} = 0.01$ m, which is much smaller than Irwin’s internal length, $\ell _{ch} = \bar{E}0 G_f / f_t^2$. The computational domain is discretized by FE meshes with the maximum size of $h_{\text {max}} = 0.5 \ell _\textrm{c}$ after the convergence test, as plotted in Fig. 4c.

**Table 2:** Parameters in our numerical simulations (Han et al. 2022; Zhang et al. 2017b)

Property	Unit	Natural gas	Cement sheath	Surrounding rock
Gas constant, *R*	J/(kg K)	520	–	–
Viscosity coefficient	Pa s	$1.133 \times 10^{-5}$	–	–
Critical pressure, $P_\textrm{c}$	Pa	$4.5 \times 10^{6}$	–	–
Critical temperature, $T_\textrm{c}$	K	185	–	–
Specific heat, $C_\textrm{p}$	J/(kg K)	3156	900	900
Specific heat, $C_\textrm{v}$	J/(kg K)	1740	–	–
Thermal conductivity, λ	W/(m K)	0.12	3.0	3.0
Thermal expansion coeff., α	1/K	–	$0.7 \times 10^{-5}$	$0.5 \times 10^{-5}$
Density, $\rho _{s}$	kg/m$^{3}$	0.716	2500	2700
Elastic modulus, *E*	Pa	–	$2.5 \times 10^{10}$	$4.0 \times 10^{10}$
Poisson’s ratio, ν	–	–	0.25	0.35
Heat transfer coefficient, *h*	W/(m$^{2}$ K)	30	–	–

### Numerical Results and Analysis

#### Gas Thermo-mechanical Coupling Responses

**Fig. 5:**
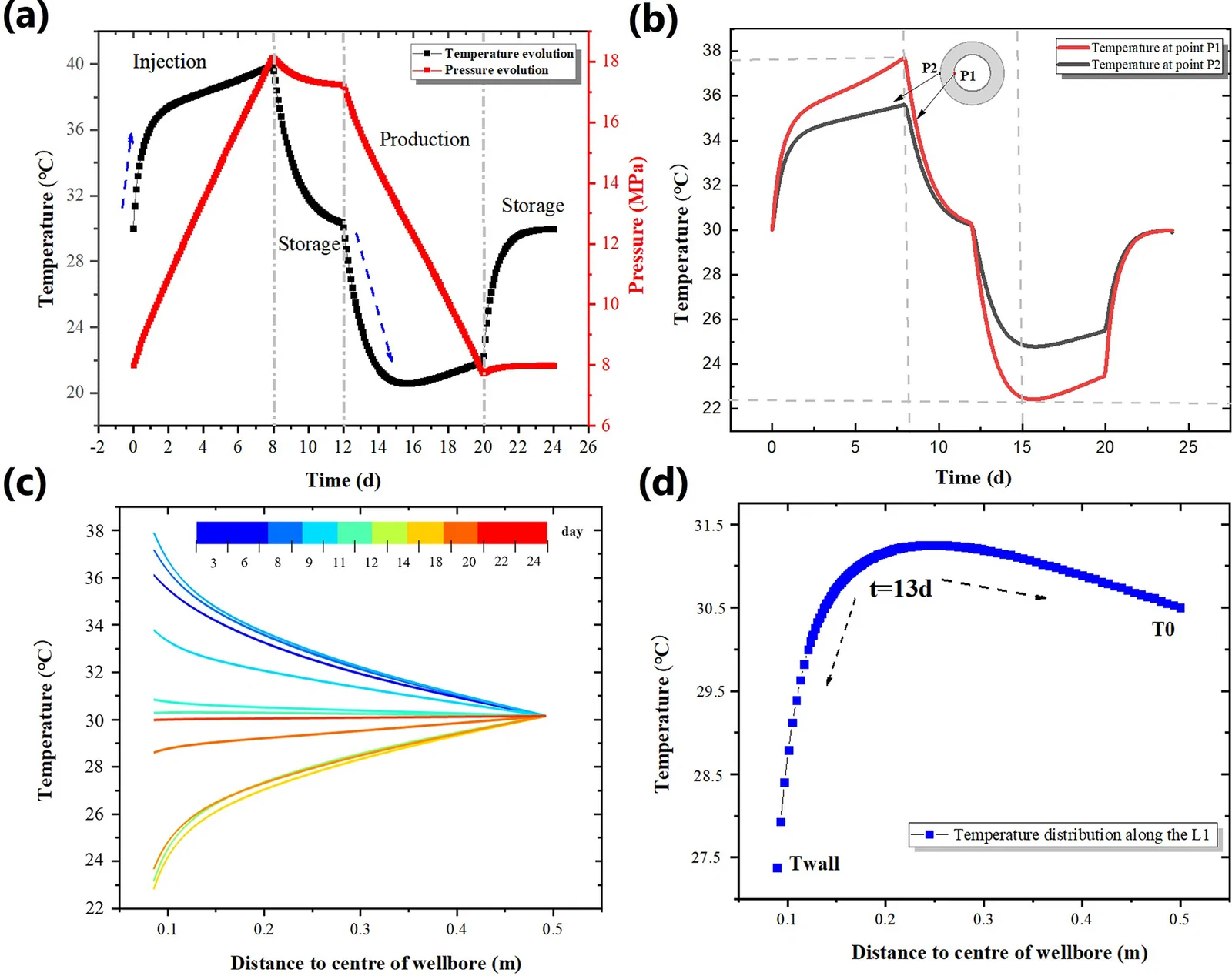
**a** Temperature and pressure variations of the gas during the injection–production cycle, **b** temperature evolution at monitoring points *P*1 and *P*2, **c** temperature distribution along monitoring line *L*1, and **d** transient cold-shock response at the wellbore wall during the early production stage

During one operation cycle, the gas temperature in the wellbore section first undergoes a rapid rise with a gradually decreasing slope, followed by a partial drop until thermal insulation is reached, as performed in Fig. 5a. This is followed by a pronounced cooling phase with a gradually increasing slope, and finally a slight rebound before stabilization. The gas pressure exhibits a similar trend to temperature but with much lower nonlinearity, appearing almost linear. The sharp temperature variations at the early stages of injection and production arise because the characteristic time scale of gas flow response is much shorter than that of heat transfer. During gas injection, the injected gas has a higher temperature, which promotes heat accumulation within the cavern. In contrast, during gas production, temperature changes are governed by both the cooling effect of gas release and heat exchange with the surrounding rock. As a result, gas temperature drops rapidly and substantially at the onset of production. At later stages, however, the gas gradually absorbs heat from the surrounding rock to re-establish thermal equilibrium, and the fluctuations diminish. Overall, as injection and production proceed, temperature variations tend to stabilize once sufficient heat transfer with the surrounding rock has occurred. The validity of our gas thermo-mechanical coupling model is confirmed by field measurements provided in the Appendix.

Figure 5b shows that temperatures at both monitoring points *P*1 and *P*2 follow the same general variation as the cavern gas. However, P1 experiences sharper fluctuations and larger peaks during both heating and cooling stages. Figure 5c illustrates that, along monitoring line L1, temperature decreases logarithmically from the high inner-wall temperature ($T_{\text {wall}}$) to the outer boundary during injection and high-pressure storage. Conversely, during production and low-pressure storage, temperature increases logarithmically from the cooled inner wall toward the formation temperature $T_0$. This behavior reflects the fundamental principle that heat transfers from regions of higher to lower temperature. At the early stage of production, the sudden gas cooling also induces a short-term cold shock on the wellbore surface, as shown in Fig. 5d. This transient thermal perturbation is critical for understanding the thermo-mechanical coupling failure mechanisms of cement sheaths.

**Fig. 6:**
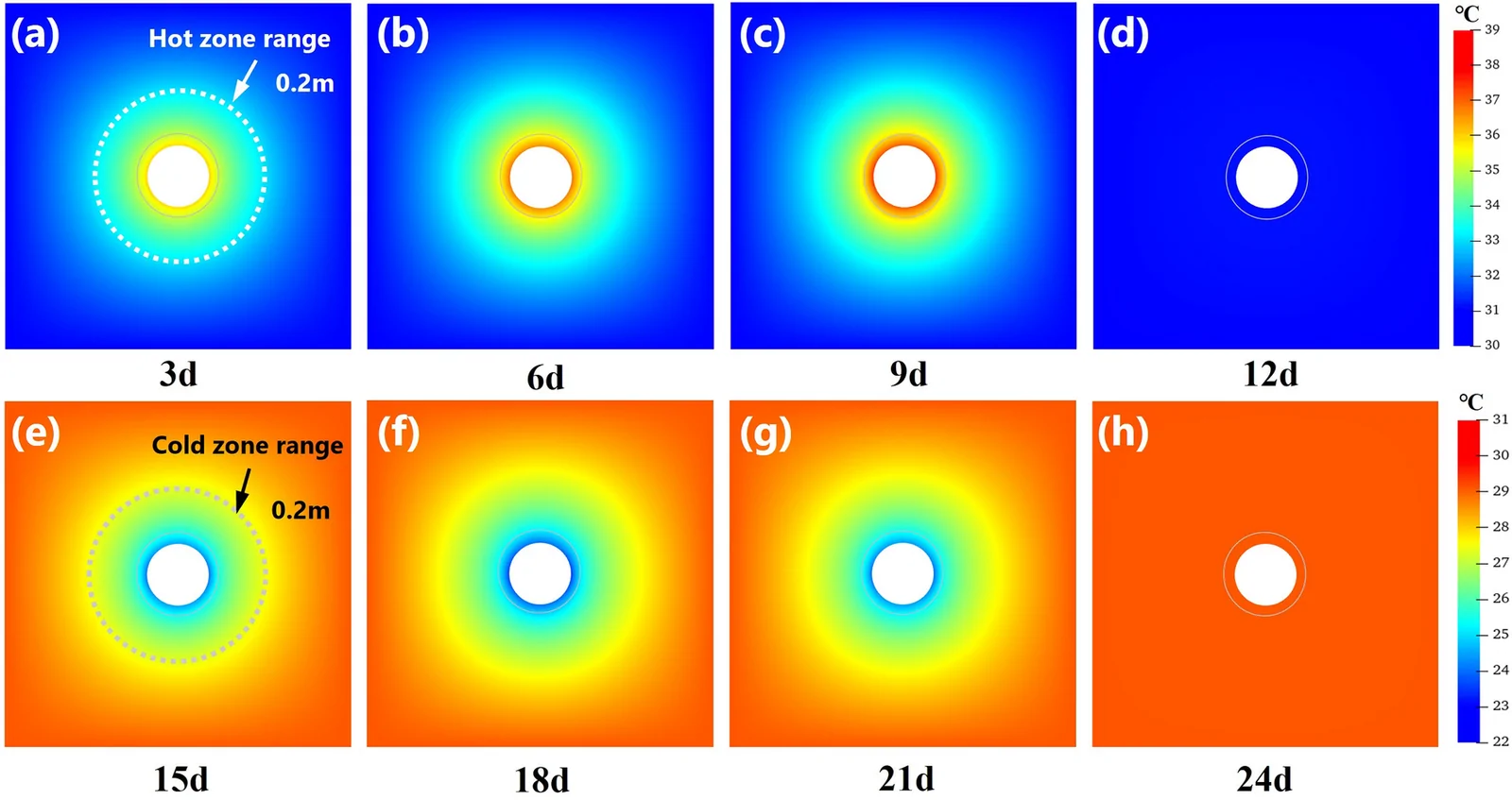
Temperature evolution of the wellbore surrounding rock during the injection stage (**a**–**d**) and the production stage (**d**–**e**)

Figure 6 shows the temperature evolution in the surrounding rock during injection and production. During injection, the gas temperature in the cavern rises because of pressurization and thermo-mechanical coupling effects. This procedure creates a heated zone on the wellbore surface (Liu et al. 2021), where strong heat transfer happens, as shown in Fig. 6a–d. During production, the gas expands and cools as the pressure decreases. This forms a cold zone around the wellbore surface, as seen in Fig. 6e–h. It can be found from Fig. 6 that both the heated and cooling zones extend about 0.20 m inside the surrounding rocks.

**Fig. 7:**
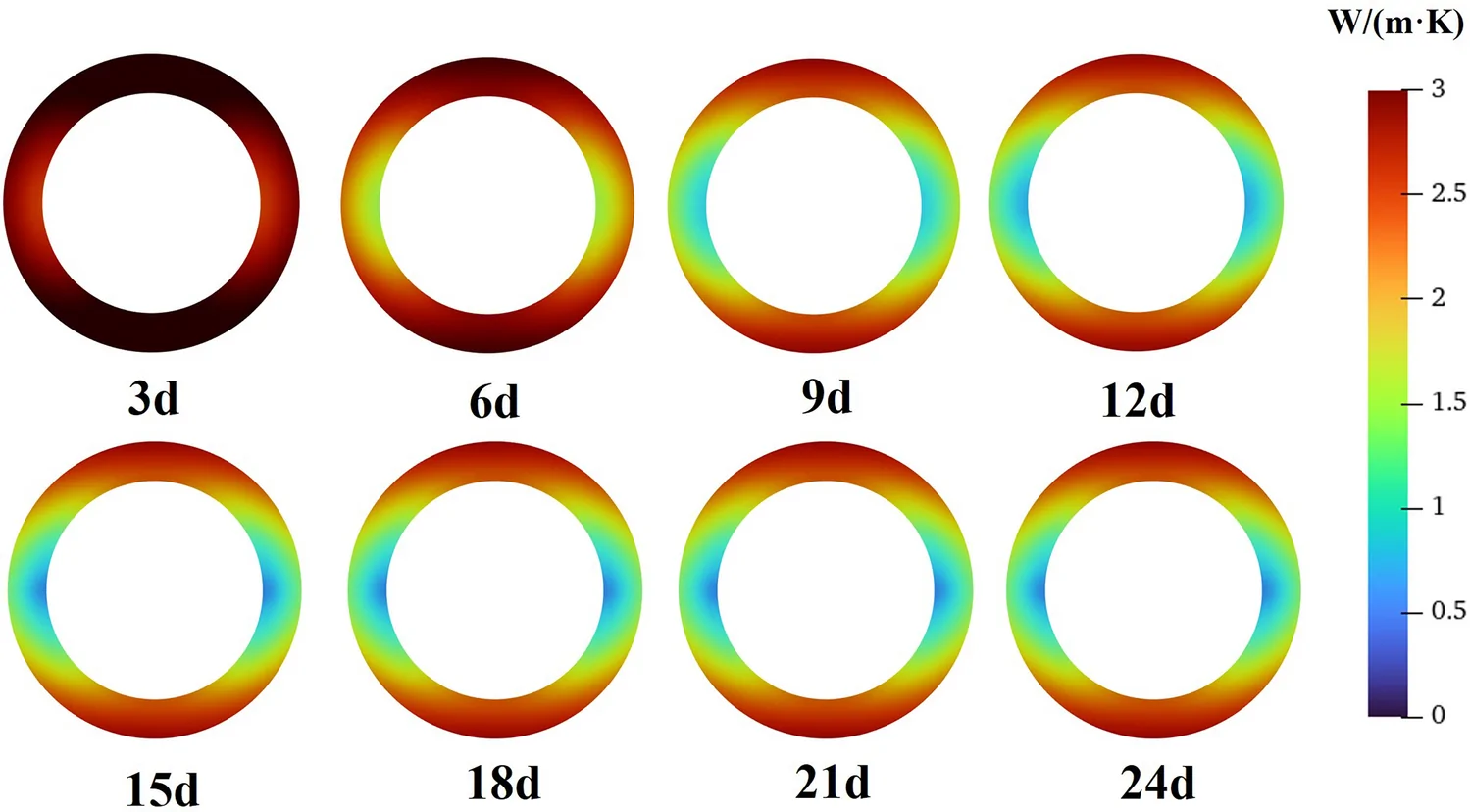
Thermal conductivity distribution of the cement sheath after damage-induced degradation

**Fig. 8:**
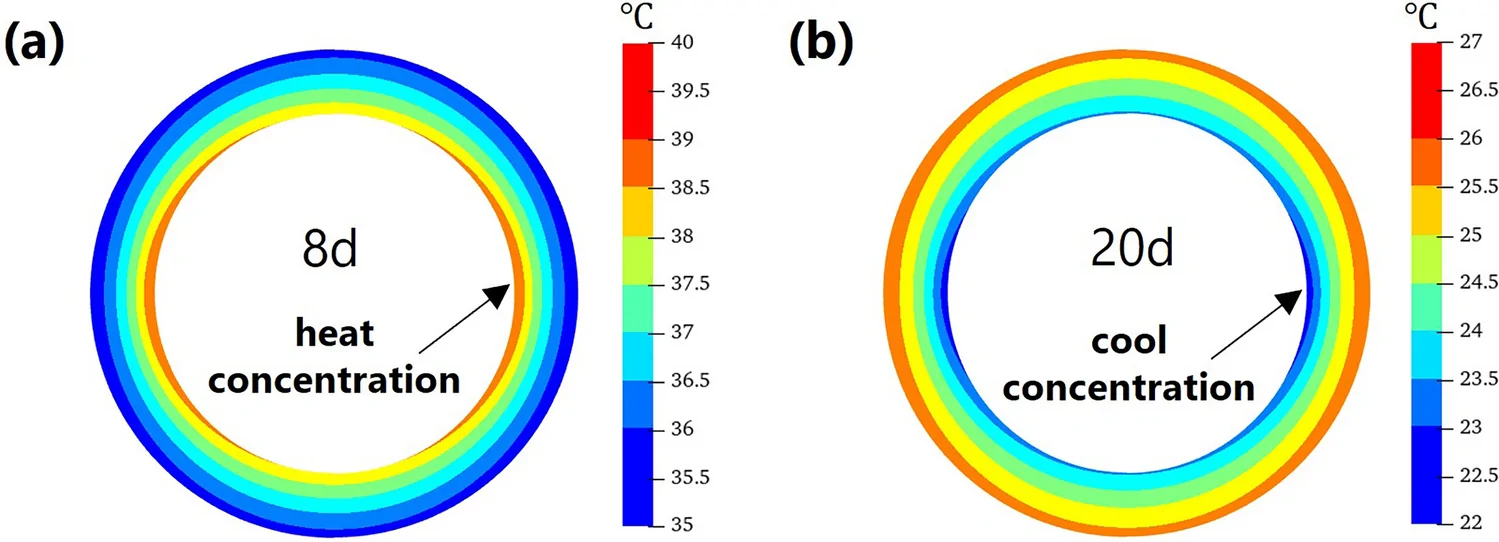
Thermal concentration and steep temperature gradients in the damaged cement sheath during the operation cycle

Once the cement sheath is damaged, the initiation, propagation and coalescence of microcracks may happen, resulting in the degradation in thermal conductivity, as shown in Fig. 7. It illustrates the evolution of thermal conductivity in the cement sheath within one injection–production cycle, showing a gradual decrease with progressive damage. Consequently, during injection–production cycles, gas may temporarily accumulate or lose heat because of the reduced heat-transfer efficiency of the damaged cement sheath. This results in abnormally high or low gas temperatures at the end of the injection and production stages, as seen in Fig. 5a–b, which may offer the underlying mechanisms of previous experimental and field observations (Liu et al. 2021; Han et al. 2022). In addition, the heat tends to concentrate or dissipate within damaged zones, leading to the concentration of temperature gradients, as indicated in Fig. 8. The concentration of temperature gradient may impose the additional thermal shocks on the cement sheath, facilitating microcracking development due to extreme thermal expansion or contraction. Furthermore, the rapid variations in gas temperature significantly affect other state parameters in the tubing, potentially intensifying erosion and corrosion, and even triggering hydrate or condensate formation (Liu et al. 2021).

#### Progressive Failure Analysis

**Fig. 9:**
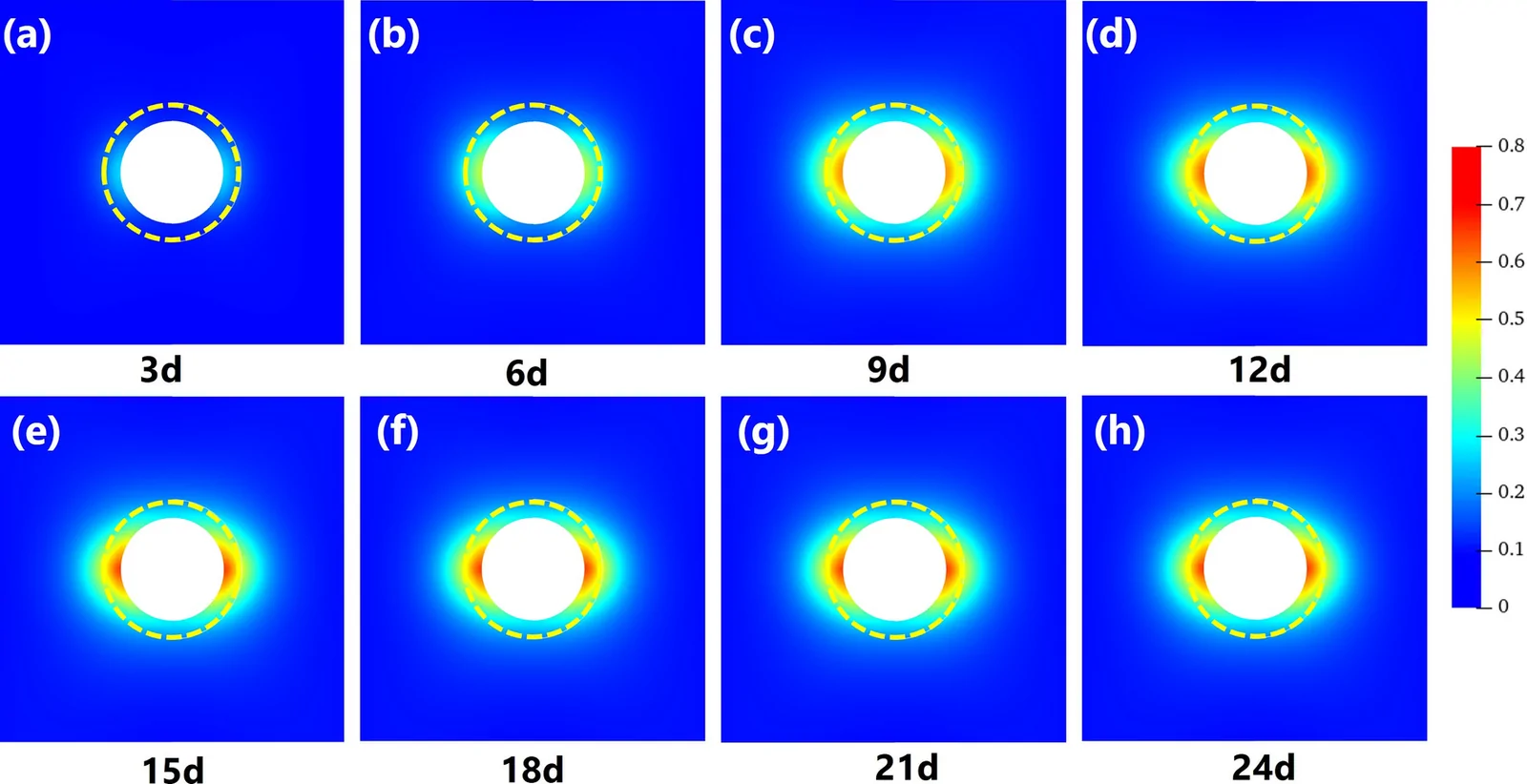
Damage evolution of the wellbore cement sheath over the complete injection–production cycle

We plot the progressive failure evolution process in the cement sheath during one injection–production cycle in Fig. 9. It can be observed from Fig. 9a, b that the slight and uniform damage first symmetrically initiates on the left and right sides at the beginning stage of natural gas injection and pressurization. From t=6 days, when the injected natural gas pressure exceeds 15.0 MPa, cracks rapidly propagate towards the inside of cement sheaths, and the maximum concentrations of crack densities are located along the horizontal direction, i.e., $\alpha = 0^\circ$ and $\alpha = 180^\circ$, as shown in Fig. 9c, d. Figure 9d, e also indicates that cracks and damage continue to develop in the cement sheath at the natural gas storage stage with the high pressure. At the beginning of gas production, transient cooling induces a cold shock that generates tensile stresses on the contracting inner surface of the cement sheath. The concentration of tensile stress interacts with the injected gas pressure and in-situ stress, which facilitates the additional damage development in wellbore cement sheaths, as shown in Fig. 9f. At the end of gas production, damage on surfaces of the wellbore cement sheath eventually reaches the maximum value of 0.743 at $\alpha = 0^\circ$ and $\alpha = 180^\circ$, as indicated in Fig. 9f. After that, Fig. 9 demonstrates that there is no additional growth of damage and microscopic cracks occurs within the same cycle.

**Fig. 10:**
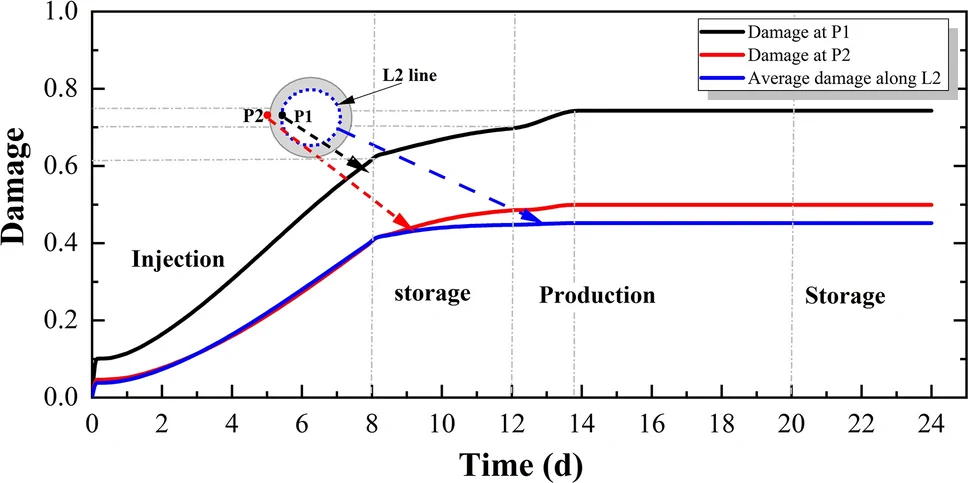
Temporal evolution of the phase-field variable ϕ (i.e., damage degree) at monitoring points $P_{1}$ and $P_{2}$ and along monitoring line $L_{2}$ in the wellbore cement sheath during a complete injection–production cycle

We also depict the temporal evolution of the damage value (i.e., phase-field variable) at two monitoring points $P_{1}$ and $P_{2}$, and the average value along the monitoring line $L_{2}$ around the inner surfaces of a cement sheath in Fig. 10. The final average value along the monitoring line $L_{2}$ around the inner surfaces of a cement sheath reaches about 0.452. The phase-field variable at the monitoring point $P_{1}$ evolves more rapidly during the gas injection and pressurization, reaching a higher peak value than that at the monitoring point $P_{2}$. Then, damage at $P_{1}$ remains nearly constant throughout the subsequent stages. In contrast, damage at $P_{1}$ increases more gradually and keeps as the constant at a lower level. Overall, most of the damage develops during gas injection and high-pressure storage, with minor transient responses during the early production stage. During the mid-to-late production and the final low-pressure storage stages, tensile damage in the cement sheath remains stable without further growth.

**Fig. 11:**
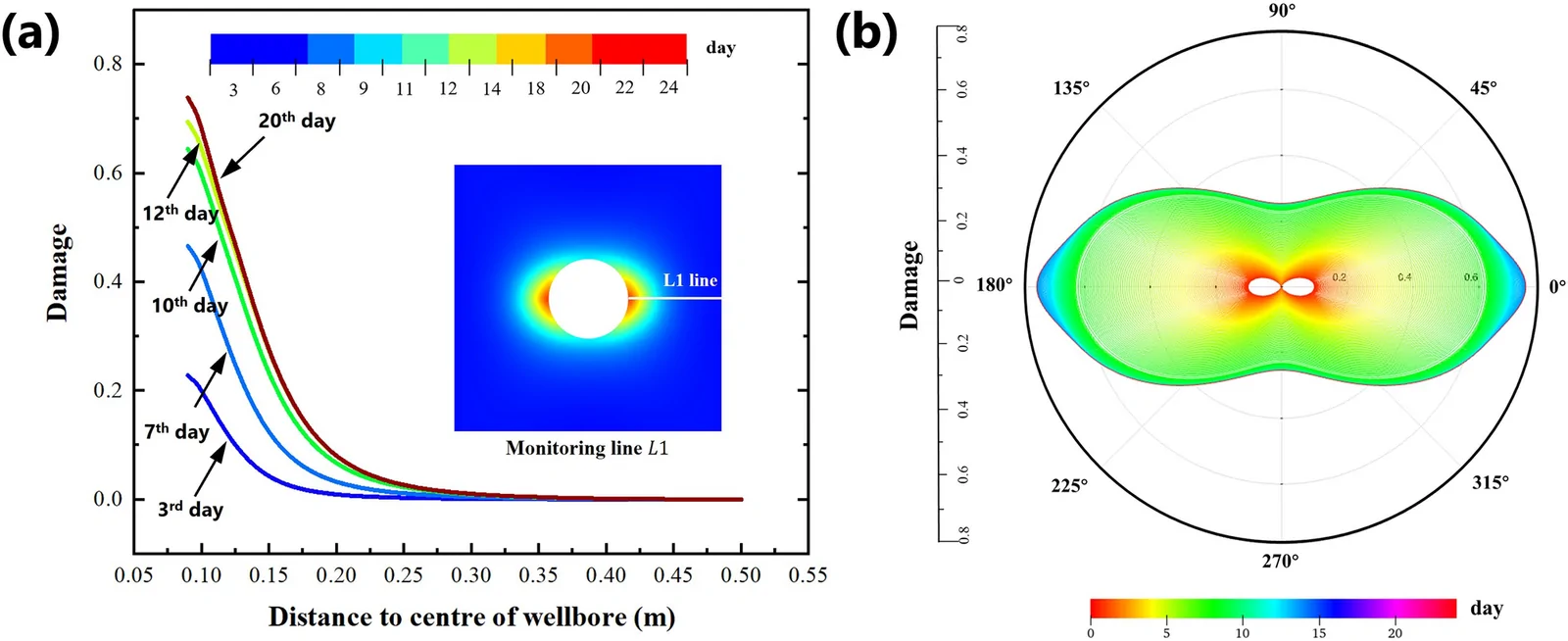
Temporal evolution and spatial distribution of the phase-field variable ϕ (i.e., damage degree) along the monitoring lines **a** $L_{1}$ and **b** $L_{2}$ in the wellbore cement sheath during a complete injection–production cycle

We further plot the temporal evolution and spatial distributions of the phase-field variable ϕ along two monitoring lines $L_{1}$ and $L_{2}$ in Fig. 11a and b, respectively. It can be observed from Fig. 11a that the damage degree/number of microcracks is mainly concentrated in the vicinity of the wellbore and gradually decreases in a nonlinear manner along monitoring line $L_{1}$. As the injection–production process progresses, the concentration of microcracks near the inner surface gradually increases. The increase in damage/microcracks during the injection stage is significantly greater than that during the production stage, as shown in Fig. 11a. Figure 11b demonstrates that damage in the wellbore cement sheath initiates symmetrically at the horizontal sides of the inner surface (i.e., $\alpha = 0^{\circ }$ and $\alpha = 180^{\circ }$) immediately after gas injection. With the increase of injection pressure, these regions experience progressive propagation and intensification of damage, forming pronounced lobes of deterioration. Meanwhile, minor damage develops at other circumferential positions, but its extent remains limited compared to the dominant horizontal zones, as shown in Fig. 11b. This distribution indicates that gas-thermally induced tensile stress concentration within the injection–production cycles dominates the preferential evolution of tensile damage/microcracks along the lateral directions of the wellbore cement sheath.

**Fig. 12:**
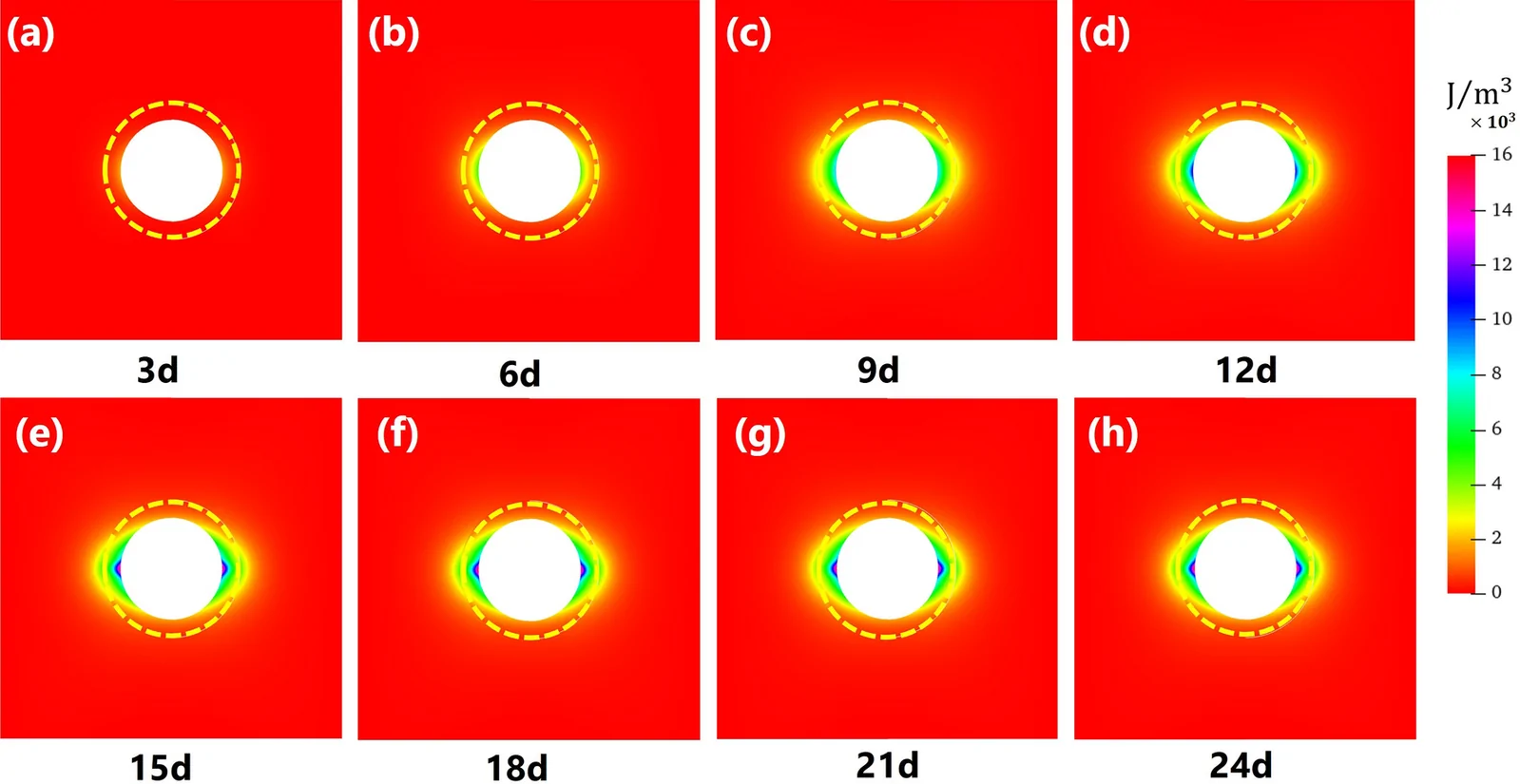
Evolution of the equivalent failure driving energy density during one injection–production cycle

Figure 12 shows the distribution of equivalent failure driving energy density $\mathcal {H}$ in our phase-field model at different stages of the operation cycle. It is observed that, starting from day 6, the equivalent failure driving energy density $\mathcal {H}$ develops and intensifies on the left and right sides of the wellbore cement sheath. The energy density concentrates around $\alpha = 0^\circ$ and $\alpha = 180^\circ$, reaching a maximum value of $16 \times 10^{3}~\mathrm {J/m^3}$. The spatial distribution and temporal evolution are consistent with the failure patterns. This numerical result further illustrates that the historical maximum positive strain energy density serves as an effective failure driving force for phase-field evolution, promoting the development of tensile damage/microcracks in the wellbore cement sheath.

**Fig. 13:**
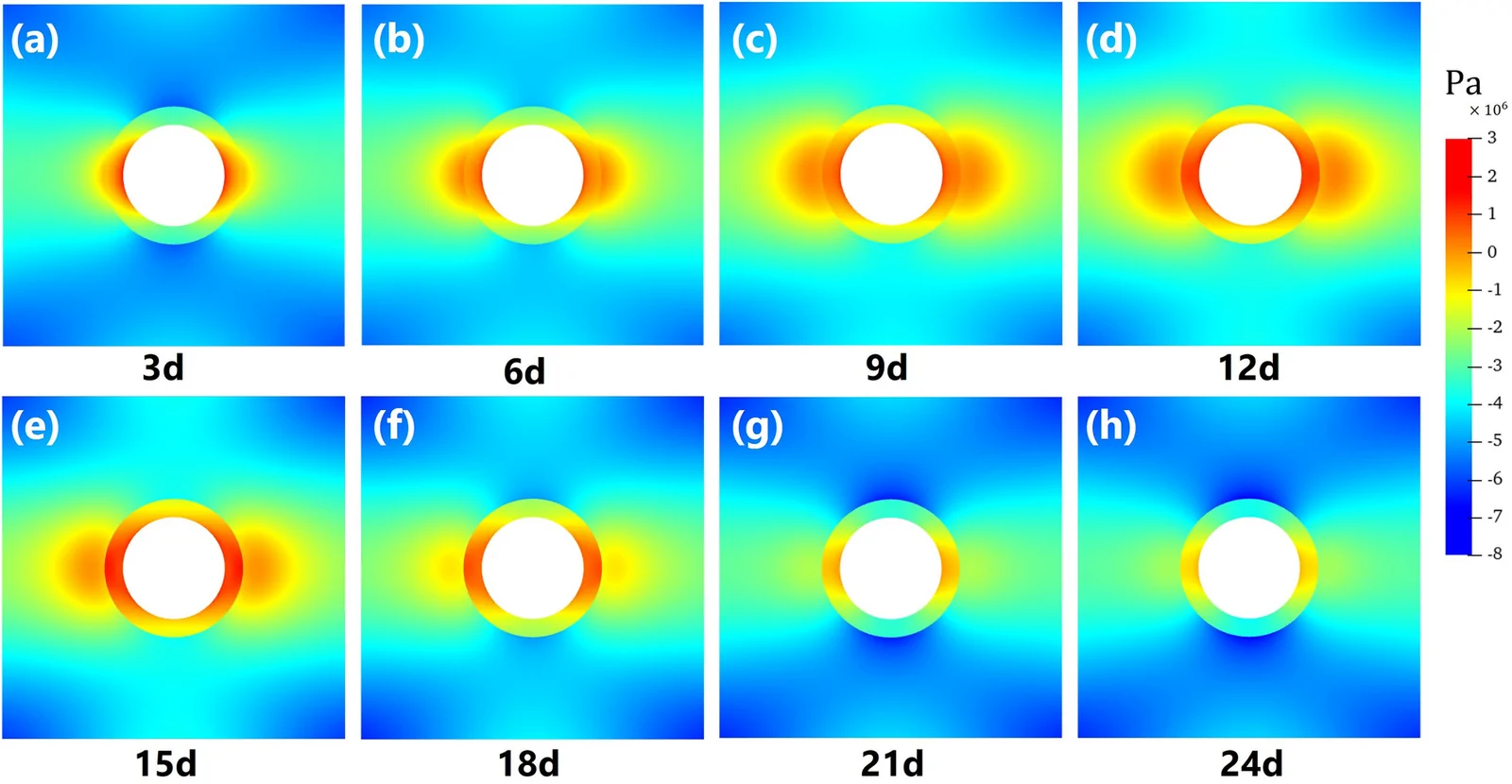
Spatiotemporal distributions of tangential stress $\sigma _{\tau }$ in the numerical model of the wellbore cement sheath during the entire injection–production cycle

**Fig. 14:**
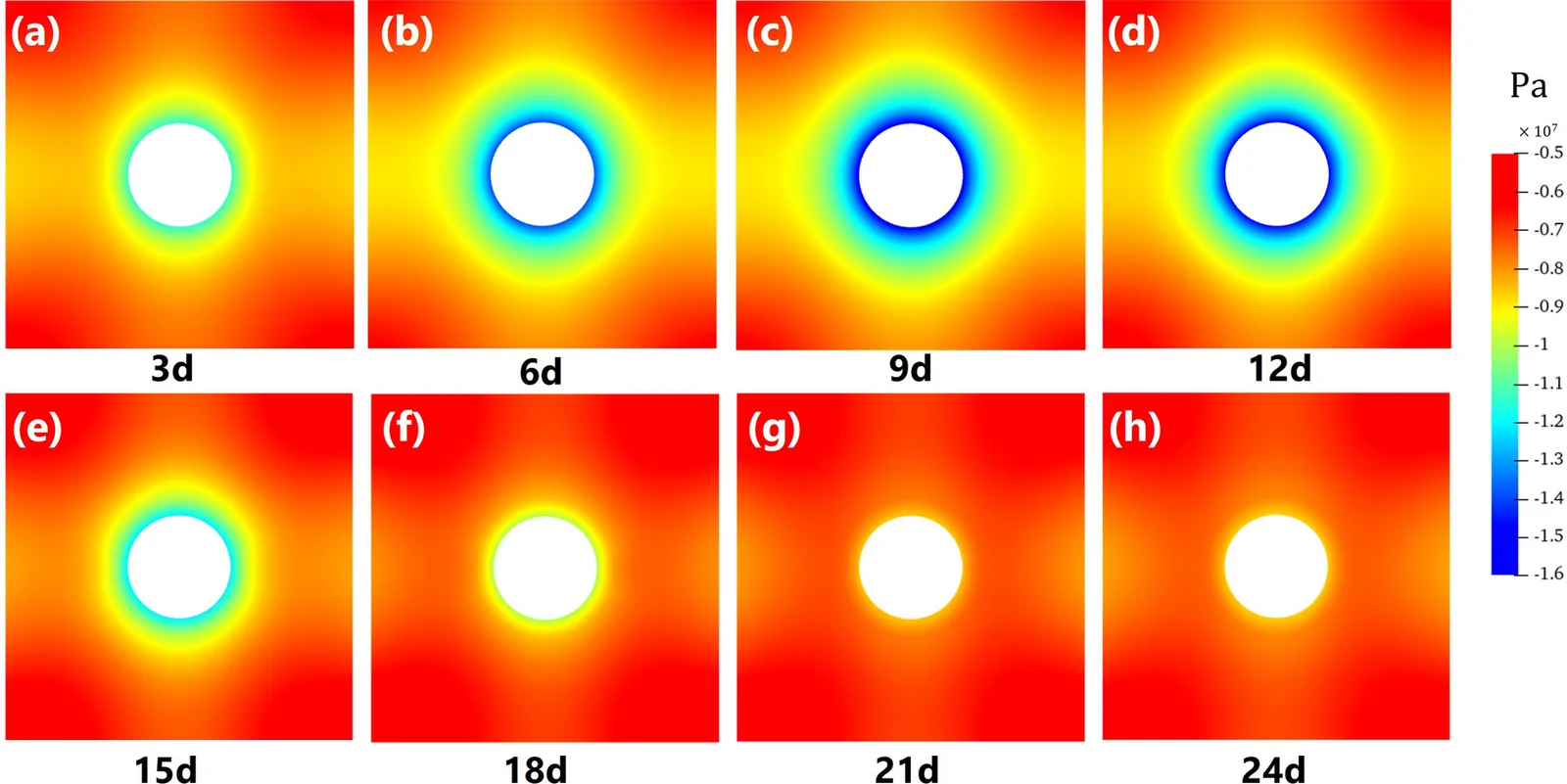
Spatiotemporal distributions of radial stress $\sigma _{r}$ in the numerical model of the wellbore cement sheath during the entire injection–production cycle

Figures 13 and 14 show the distributions of tangential and radial stresses around the wellbore cement sheath during the operation cycle. From the gas injection stage to high-pressure storage, the cement sheath transitions from tangential compression at $\alpha = 90^\circ$ and $\alpha = 270^\circ$, and tangential tension at $\alpha = 0^\circ$ and $\alpha = 360^\circ$, to tensile stress in all tangential directions due to the combined effects of in-situ stress and gas pressure. The maximum tangential tensile stress is approximately 3.0 MPa, while the maximum radial compressive stress reaches 18 MPa. These results are consistent with the analytical solution from elasticity theory, which states that stresses around a circular hole in a biaxially compressed plate with internal pressure are given by the superposition of far-field stress and internal pressure. In this case, the maximum gas pressure reaches 18.0 MPa, whereas the maximum geostress is 9.1 MPa. The tangential stress concentration factor under biaxial loading is twice the applied far-field stress, while under internal pressure alone it equals the internal pressure itself. Based on this superposition principle, the analytical tangential stress in the cement sheath is about 3.0 MPa, which agrees with the numerical results.

Over time, damage initiates and evolves from the inner surface of the cement sheath. Some microscale circumferential tensile cracks may arrest due to stress dissipation effects, whereas the dominant damage zones at $\alpha = 0^\circ$ and $\alpha = 360^\circ$ continue to propagate into macroscopic cracks. Tangential stress, acting as the maximum principal stress $\sigma _{1}$, concentrates at the crack tips. This behavior is consistent with fracture mechanics theory and indicates that macroscopic cracks form by tensile failure. Moreover, the maximum principal stress decreases with increasing phase-field values, and tangential stresses at the inner surface gradually transition from tension to compression. The tensile stresses inside the cement sheath are transferred outward, becoming tangential tensile stresses in the far field. This occurs because injected gas penetrates damaged cracks after sheath failure, applying compressive loads to the crack surfaces.

In addition, the damaged wellbore cement sheath exhibits considerable degradation in thermal conductivity. During the initial stages of gas production or after repeated operation cycles, temperature fluctuations become more pronounced, which in turn affects gas pressure, causing it to deviate either upward or downward. In the following sections, we will discuss the thermo-mechanical coupling effects on the evolution of damage/microcracks in the wellbore cement sheath, particularly with the respect to the enhanced rates of injection and production during operations. Gas injection heats the inner surface of the cement sheath, while subsequent cooling promotes annular tensile damage along the inner surface. Under the fully thermo-mechanical coupling conditions, the damaged cement sheath may further deteriorate during new operation cycles.

## Numerical Discussions and Applications

### Parametric Study and Sensitivity Analysis

In this section, we conduct a series of numerical simulations on the wellbore cement sheath under various operational scenarios to investigate how in-situ stress, the critical fracture-energy release rate, cement-sheath elastic modulus, and gas injection–production rates influence failure modes and the critical operating pressure. Note that a safety threshold of 0.5 in wellbore cement sheath is adopted to determine the critical operating pressure, i.e., $\max \phi \le 0.5$ is taken to indicate no significant macroscopic cracking. Thermo-mechanical coupling parameters and material properties follow those listed in Sect. 4.1; geometric and boundary conditions are kept the same.All simulations in this section are performed based on Sect. 4, with the results being both qualitative and quantitative.

#### Effect of In-Situ Stress State

**Fig. 15:**
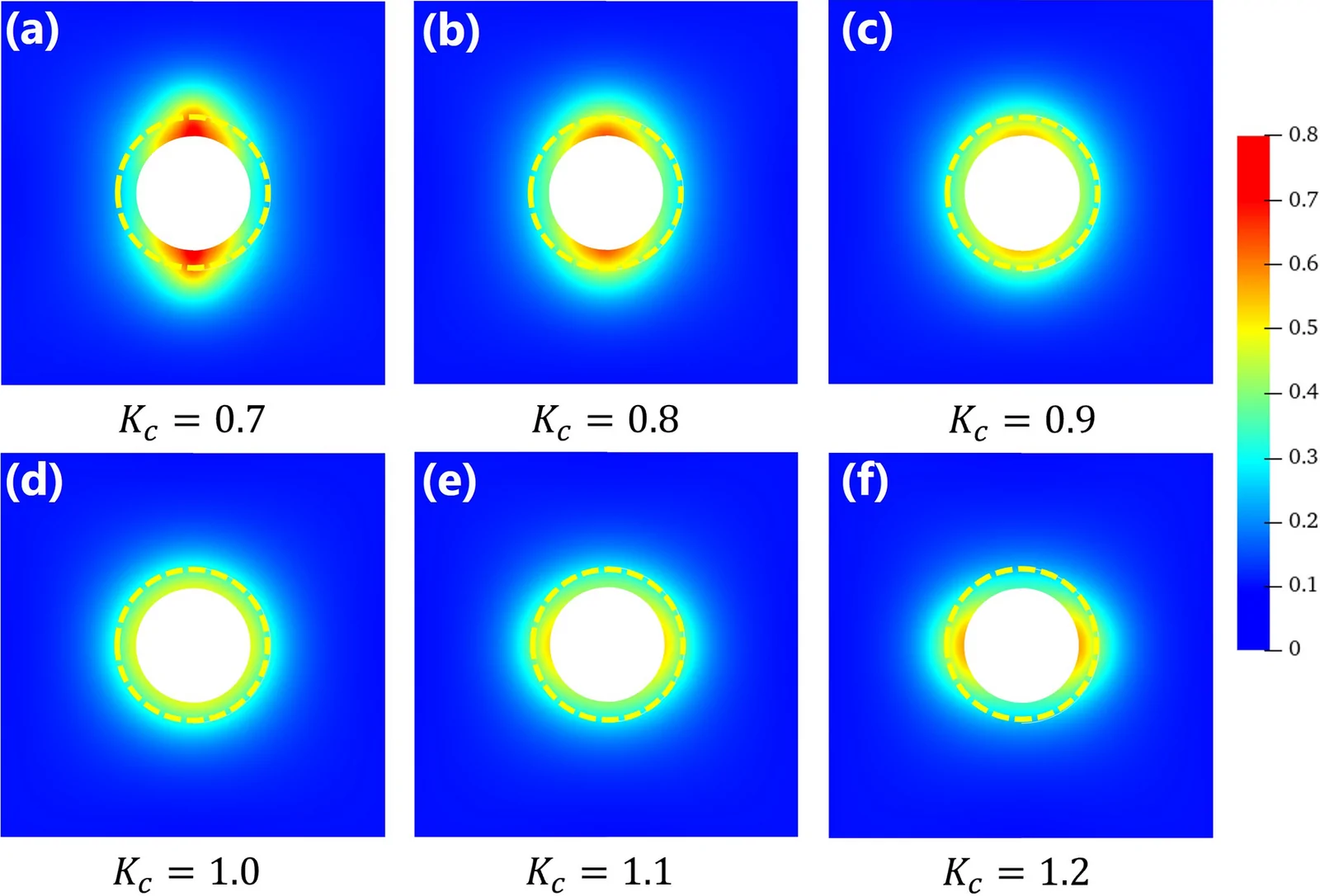
Ultimate failure patterns of the wellbore cement sheath subjected to different lateral pressure coefficients $K_\textrm{c}$ over one complete injection–production cycle

In deep rock engineering, the in-situ stress state plays a critical role, as it governs the stability of underground excavations and boreholes (He et al. 2024; Topcu et al. 2024; Zou and Lin 2024). To investigate the effect of lateral in-situ stress on the failure patterns of wellbore cement sheaths, we simulated six numerical samples under different lateral pressure coefficients $K_\textrm{c}$, ranging from 0.7 to 1.2 in increments of 0.1, over one complete injection–production cycle. The ultimate failure patterns are shown in Fig. 15a–f.

**Fig. 16:**
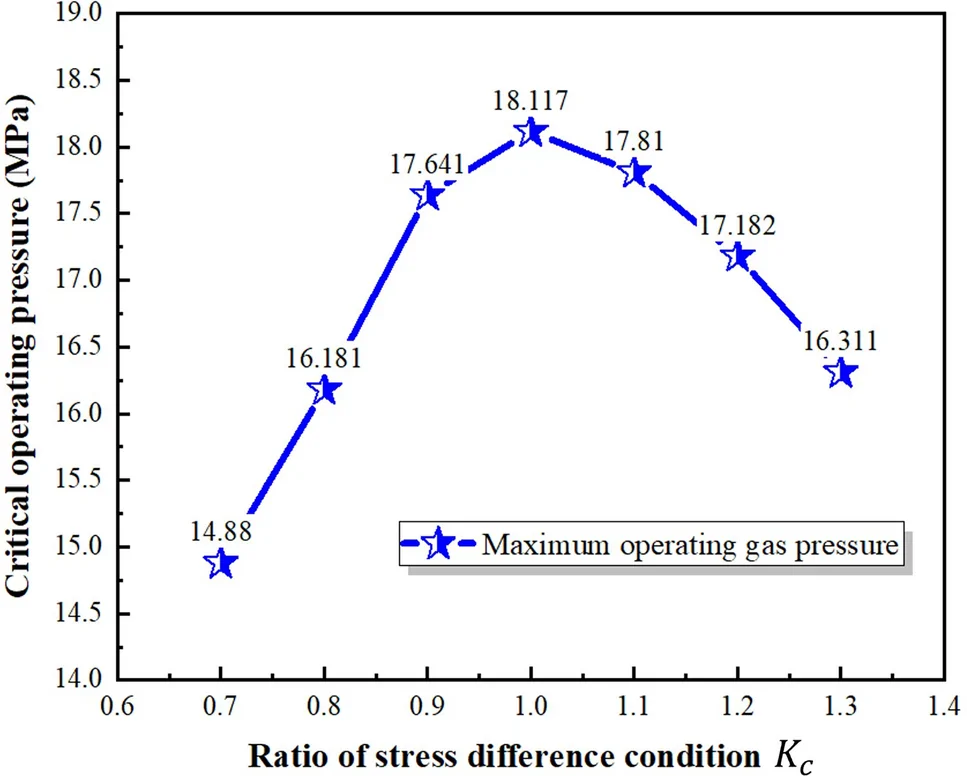
Relationship between the lateral pressure coefficient $K_\textrm{c}$ and the critical operating pressure of salt cavern gas storage, showing the maximum allowable internal pressure under different stress conditions

The results indicate that for $K_\textrm{c} = 0.7$, 0.8, and 0.9, tensile damage is concentrated at $\alpha = 90^\circ$ and $\alpha = 270^\circ$, whereas for $K_\textrm{c} = 1.1$ and 1.2, damage localizes at $\alpha = 0^\circ$ and $\alpha = 180^\circ$. At $K_\textrm{c} = 1.0$, corresponding to isotropic stress conditions, uniform circumferential damage develops along the cement sheath. The most severe damage occurs at $K_\textrm{c} = 0.7$, with a maximum damage value of 1.0, while the least damage is observed at $K_\textrm{c} = 1.0$, with a maximum value of 0.5. A safety threshold of 0.5 is adopted to determine the critical operating pressure (i.e., $\max \phi \le 0.5$, no significant macroscopic cracks). As shown in Fig. 16, the critical pressure reaches a maximum of approximately 18.12 MPa at $K_\textrm{c} = 1.0$ and decreases to 14.88 MPa at $K_\textrm{c} = 0.7$. Overall, tensile failure induced by stress anisotropy markedly compromises wellbore integrity.

#### Effect of Critical Fracture Energy Release Rate

**Fig. 17:**
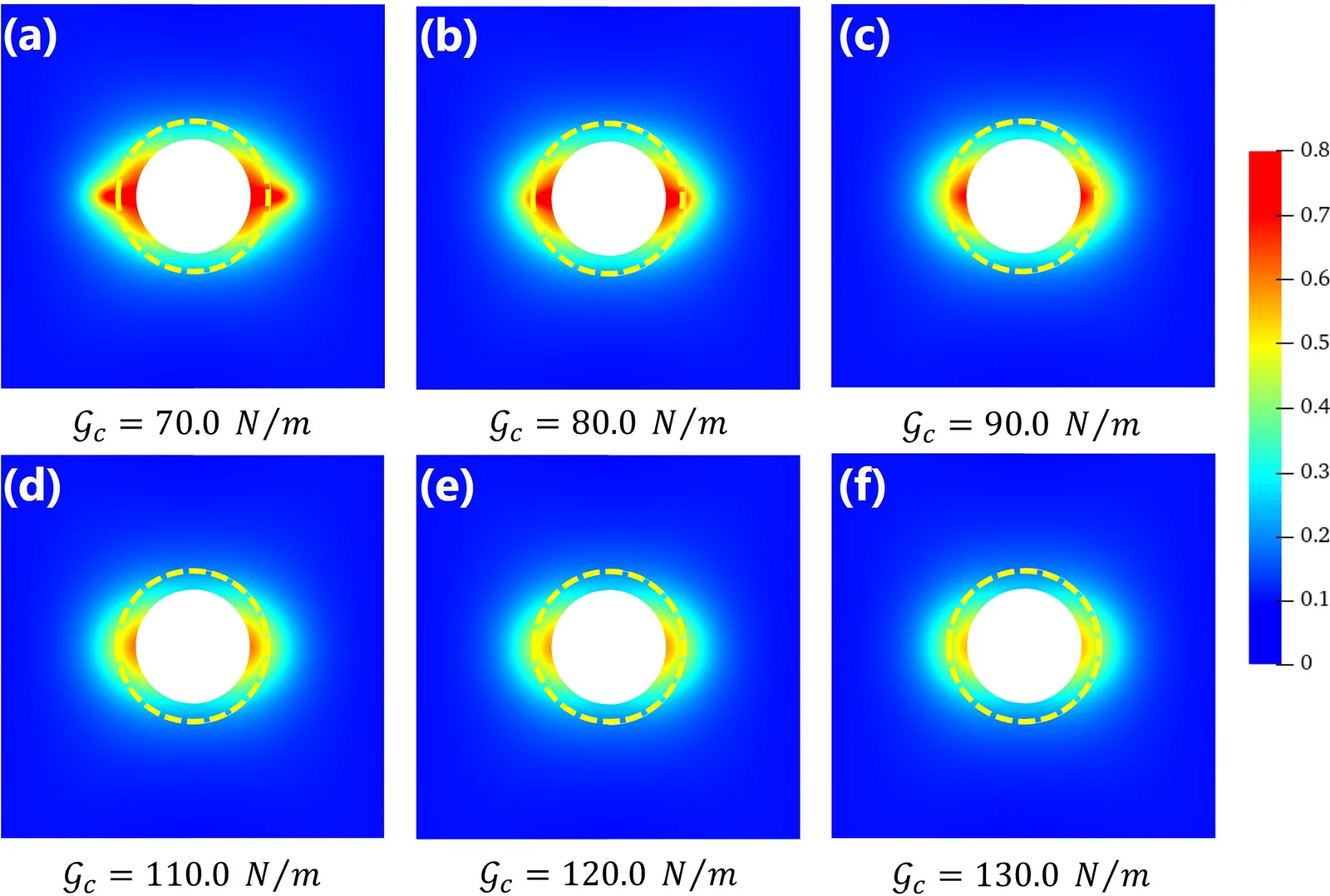
Ultimate failure patterns of the wellbore cement sheath with various critical fracture energy release rates $\mathcal {G}_\textrm{c}$ subjected to one complete injection–production cycle

**Fig. 18:**
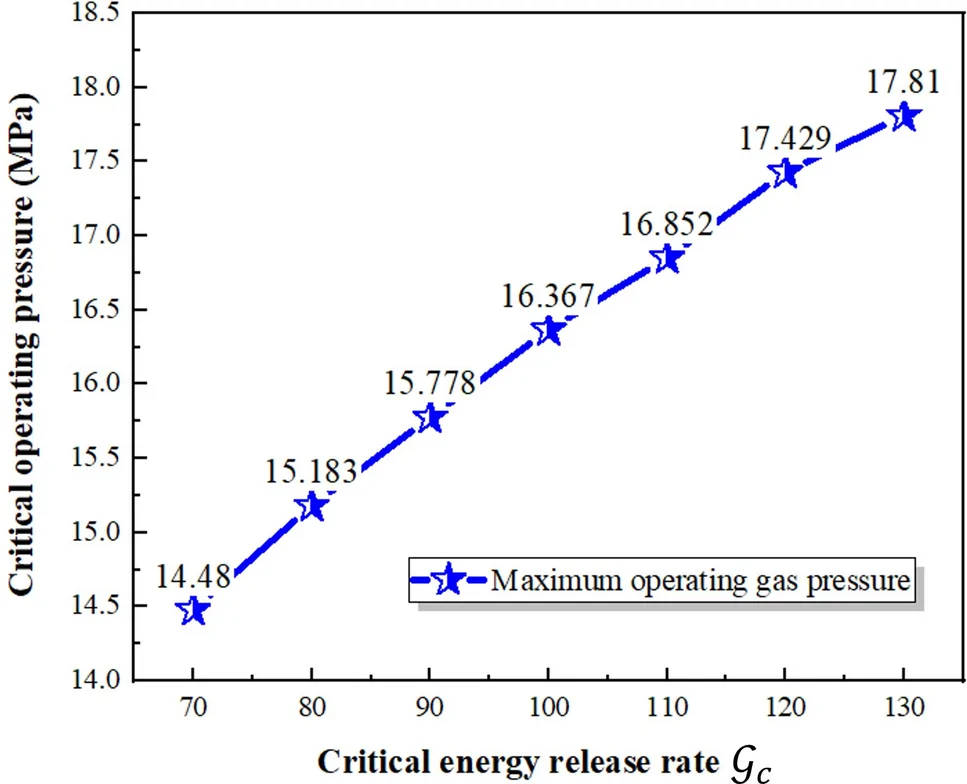
The maximum allowable internal pressure, i.e., critical operating gas pressure, on cement sheaths with various critical fracture energy release rates $\mathcal {G}_\textrm{c}$

To study the effect of critical fracture energy release rate $\mathcal {G}_\textrm{c}$ in cement materials, we simulated six different numerical samples with various critical fracture energy release rates. Figure 17 illustrates the final failure patterns of the cement sheath with different critical fracture energy release rates $\mathcal {G}_\textrm{c}$. The numerical results indicate that variations in $\mathcal {G}_\textrm{c}$ have only a minor effect on the overall failure mode. In all cases, failure initiates at $\alpha = 0^\circ$ and $\alpha = 180^\circ$ along the inner surface of the cement sheath and subsequently propagates outward. However, the severity of failure is strongly affected by the magnitude of $\mathcal {G}_\textrm{c}$. A smaller $\mathcal {G}_\textrm{c}$ leads to more pronounced failure development, reflecting the increased fracture resistance of the material. As shown in Fig. 18, the corresponding critical operating pressure of the storage wellbore exhibits an approximately linear increase with $\mathcal {G}_\textrm{c}$. The maximum critical operating pressure reaches 17.81 MPa when $\mathcal {G}_\textrm{c} = 130$ N/m, whereas the minimum value of 14.48 MPa is obtained at $\mathcal {G}_\textrm{c} = 70$ N/m. This trend arises because a higher $\mathcal {G}_\textrm{c}$ corresponds to greater resistance against crack initiation and propagation, thereby enhancing the wellbore’s capacity to sustain higher internal pressures.

#### Effect of Elastic Modulus of Cement Sheaths

**Fig. 19:**
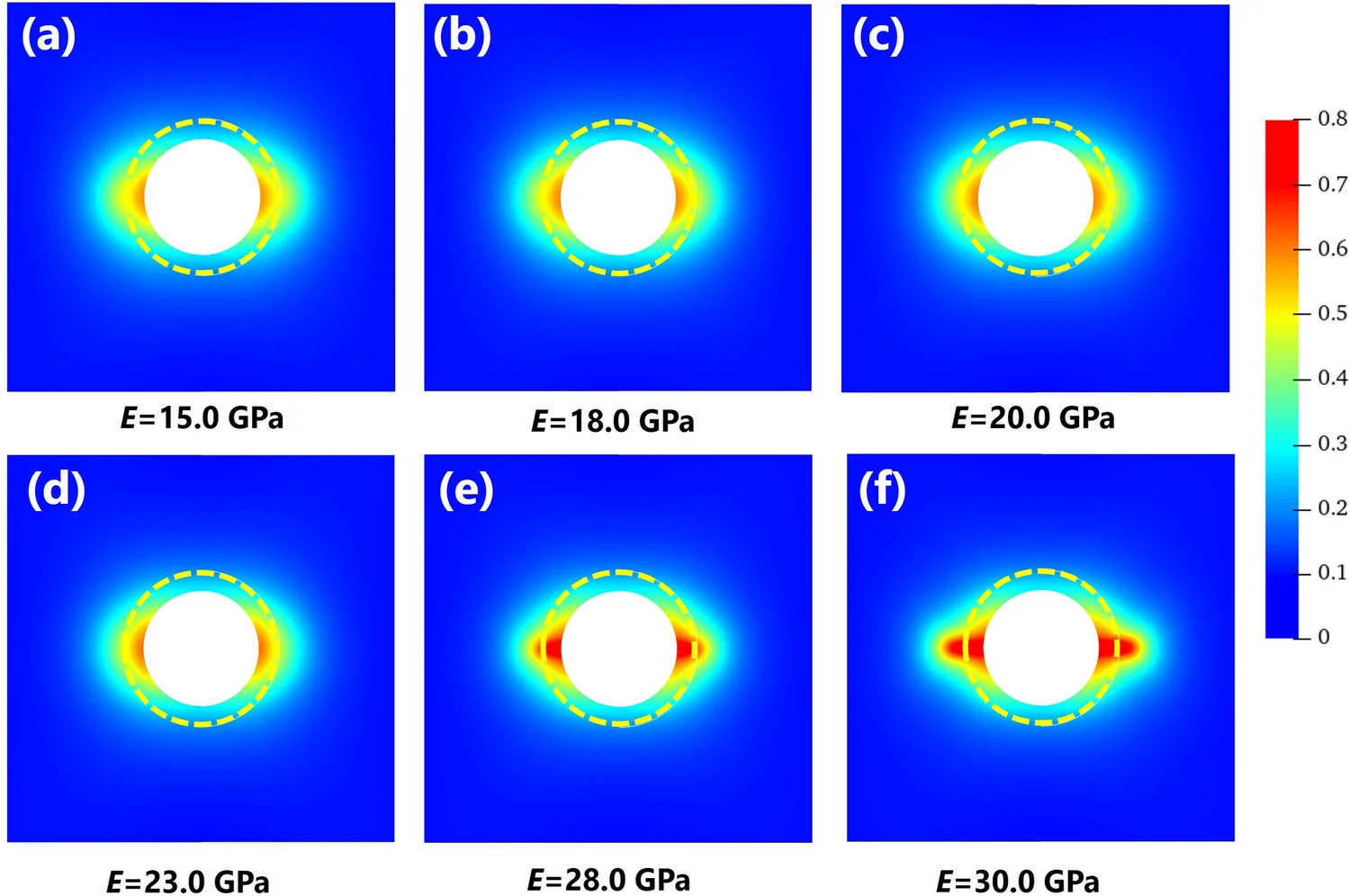
Ultimate failure patterns of the wellbore cement sheath with different elastic moduli *E* subjected to one complete injection–production cycle

**Fig. 20:**
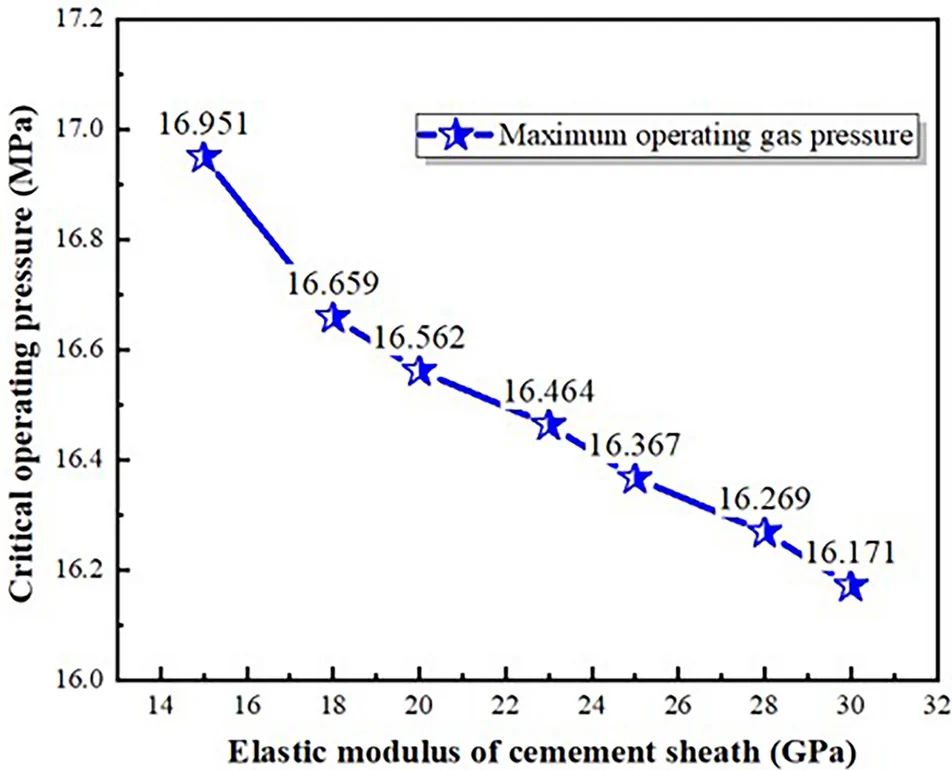
Relationship between the critical operating pressure of salt cavern gas storage and the elastic modulus of the wellbore cement sheath

Furthermore, we also explore the effect of elastic modulus of cement sheaths on the ultimate failure patterns after one complete injection–production cycle using a series of simulations. We set the elastic modulus of cement sheaths as E=15.0 GPa, E=18.0 GPa, E=20.0 GPa, E=23.0 GPa, E=28.0 GPa and E=30.0 GPa. Numerical results of the ultimate failure patterns of these six numerical wellbore cement sheaths with various elastic moduli are presented in Fig. 19a–f, respectively. For lower elastic moduli (*E* = 15.0–20.0 GPa), failure initiates at $\alpha = 0^\circ$ and $\alpha = 180^\circ$ along the inner surface, with limited propagation and relatively small affected zones. At E=23.0 GPa, the failure intensity increases, with wider localized zones developing along the same angular positions. When the modulus increases further to E=28.0 and 30.0 GPa, failure becomes significantly more severe, with extensive propagation and higher concentration around the inner wall, particularly at $\alpha = 0^\circ$ and $\alpha = 180^\circ$. Across all cases, the location of failure initiation remains consistent, while the extent of failure progressively enlarges with increasing stiffness. The corresponding critical operating pressures, shown in Fig. 20, decrease systematically with higher elastic modulus. The maximum critical pressure of 16.95 MPa is obtained at E=15.0 GPa, while values fall below 15.5 MPa at E≥28.0 GPa.

#### Effect of Injection–Production Rates

**Fig. 21:**
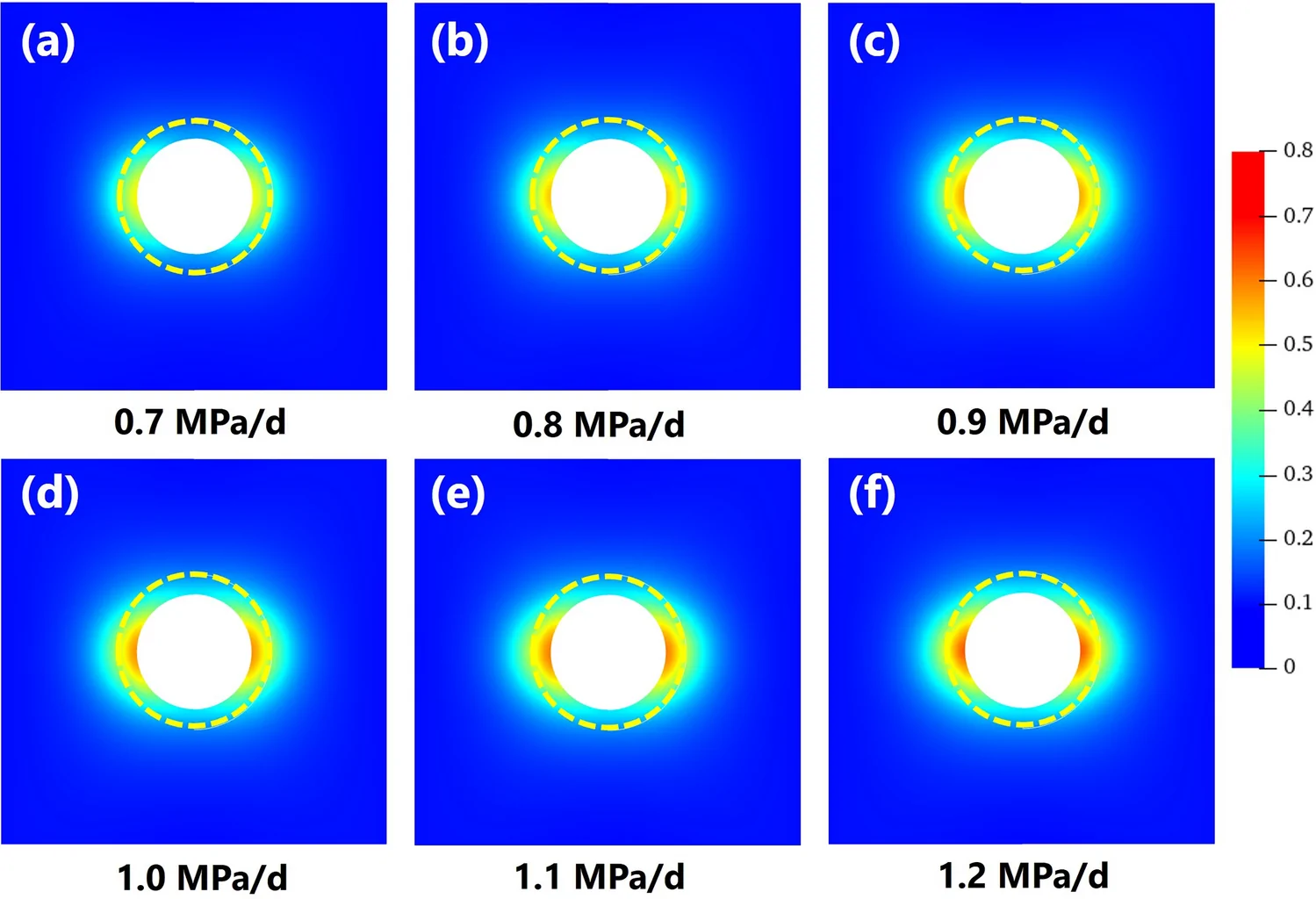
Ultimate failure patterns of the wellbore cement sheath subjected to one complete injection–production cycle with different rates

During gas injection and production, the wellbore is subjected to dynamically evolving temperature and pressure fields. These transient conditions directly alter the stress state, pore pressure, and thermo-hydro-mechanical (THM) response of the wellbore structure. To examine the associated failure mechanisms in the cement sheath, seven injection/production rates ranging from 0.7 to 1.3 MPa/day were analyzed. As shown in Fig. 21, the overall failure mode remains similar across different rates; however, at the higher rate of 1.3 MPa/day, failure initiates earlier and propagates more rapidly compared with lower rates.

**Fig. 22:**
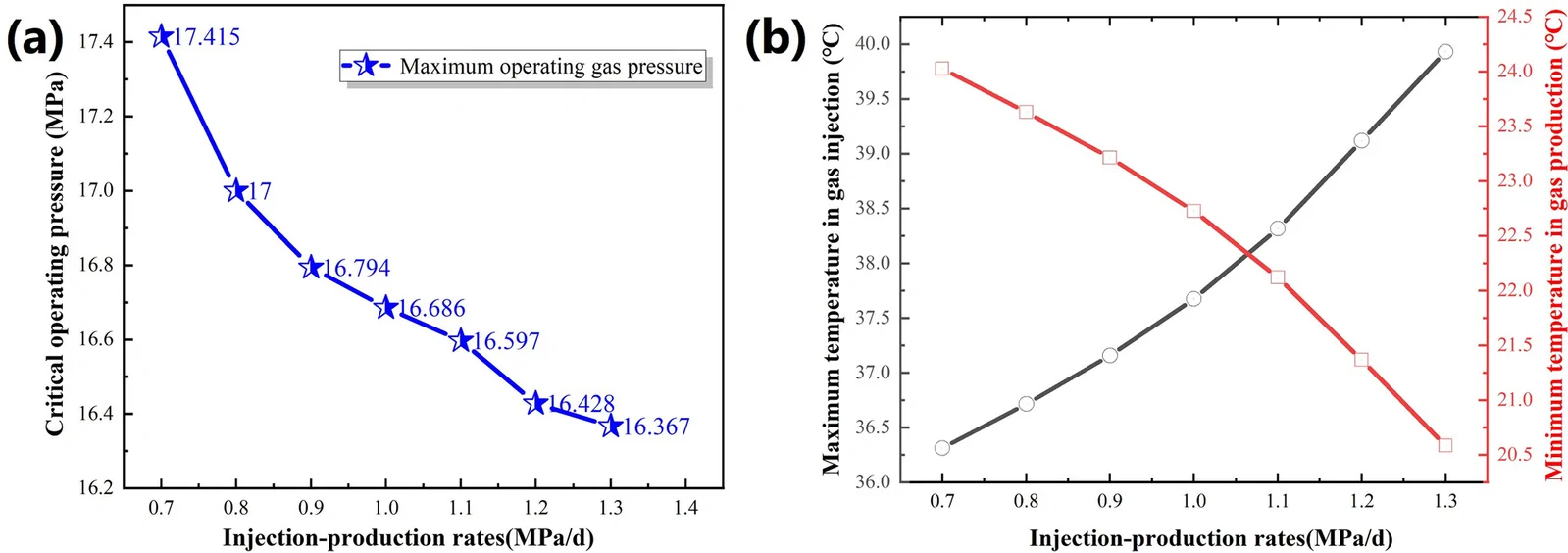
**a** Critical operating pressure of the salt cavern gas storage during injection–production cycles at different operation rates. **b** Maximum gas temperature at the end of injection and minimum temperature at the beginning of production for varying operation rates

This leads to a reduction in the critical operating pressure, as presented in Fig. 22a. The increased susceptibility at high rates is attributed to rapid stress fluctuations in the cement sheath, which generate shock-like responses. Moreover, faster injection and production intensify temperature variations, causing thermal shocks that further promote failure. As illustrated in Fig. 22b, the gas temperature peaks at the end of injection and decreases sharply during the early production stage, with the cement sheath exhibiting a similar trend. These fluctuations exacerbate tensile thermal cracking along the inner surface and trigger compressive shear spalling. Overall, higher injection/production rates accelerate the concentration of thermal stresses, thereby expediting the evolution of failure in the cement sheath.

From the above analysis, it can be found that microcracking evolution in the cement sheath is strongly dependent on the injection–production rates. At low operating rates, microcrack growth remains stable and grows gradually, resulting in the maintenance of wellbore integrity. At moderate rates, unstable propagation and partial coalescence of cracks may happen, but the wellbore structure remains relatively secure. At high rates, the unstable crack propagation and extensive crack coalescence pose a serious threat to wellbore integrity. Considering thermo-mechanical coupling effects, the low injection rates accompanied with slow temperature variation and reduced thermal stresses will lead to the higher critical operating pressures. Conversely, high injection–production rates induce sharp temperature fluctuations, which amplify thermal stresses and significantly accelerate failure.

### Field Investigations and Applications

Significant variations in geostress and geothermal gradients occur along the depth of a wellbore. At depths of several thousand meters, the geostress at the wellbore bottom can reach tens of megapascals, whereas near the wellhead it may be only a few pascals. Similarly, the geothermal temperature difference between the bottom and the wellhead can span several tens of degrees Celsius. By contrast, gas storage wells typically operate as single-opening systems, and the pressure and temperature differences of the stored gas between the bottom and the wellhead are relatively small—generally within a few megapascals and a few degrees Celsius (Han et al. 2022). Understanding cement sheath failure at different wellbore depths is therefore essential for a quantitative integrity assessment. It enables more accurate prediction and prevention of severe failures such as spalling, surface collapse, leakage, blowouts, and equipment damage. In this work, we examined the wellbore integrity at depths of 150–1000 m using field-calibrated gas pressure and temperature boundary conditions.

**Fig. 23:**
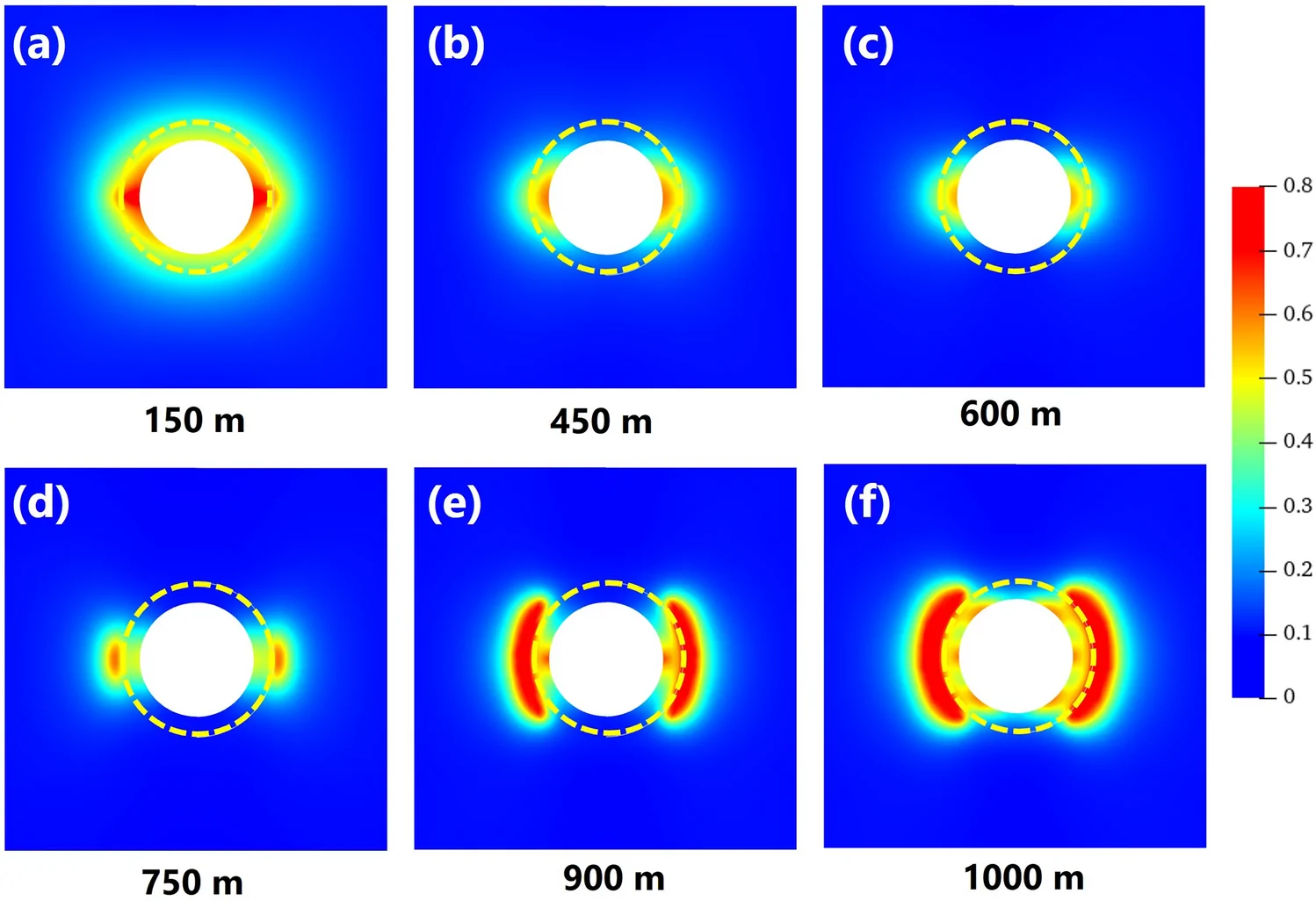
Ultimate failure patterns of the wellbore cement sheath at different depths in salt cavern gas storage

As shown in Fig. 23, the cement sheath near the wellhead exhibits a higher risk of tensile failure during gas injection compared with the bottom of the wellbore. At greater depths, high geostresses promote interfacial failure between the cement sheath and surrounding rock, leading to circumferential cracking. Overall, the wellhead region is characterized by low geostress but a high risk of tensile failure during gas injection and high-pressure storage, whereas the bottom region is dominated by high geostress and complex multiphysical couplings. When we consider the anisotropic properties of rock materials, the failure patterns in this region would likely become more complex. The comparison shows that the middle section of the wellbore demonstrates relatively greater integrity than both the wellhead and bottom sections.

**Fig. 24:**
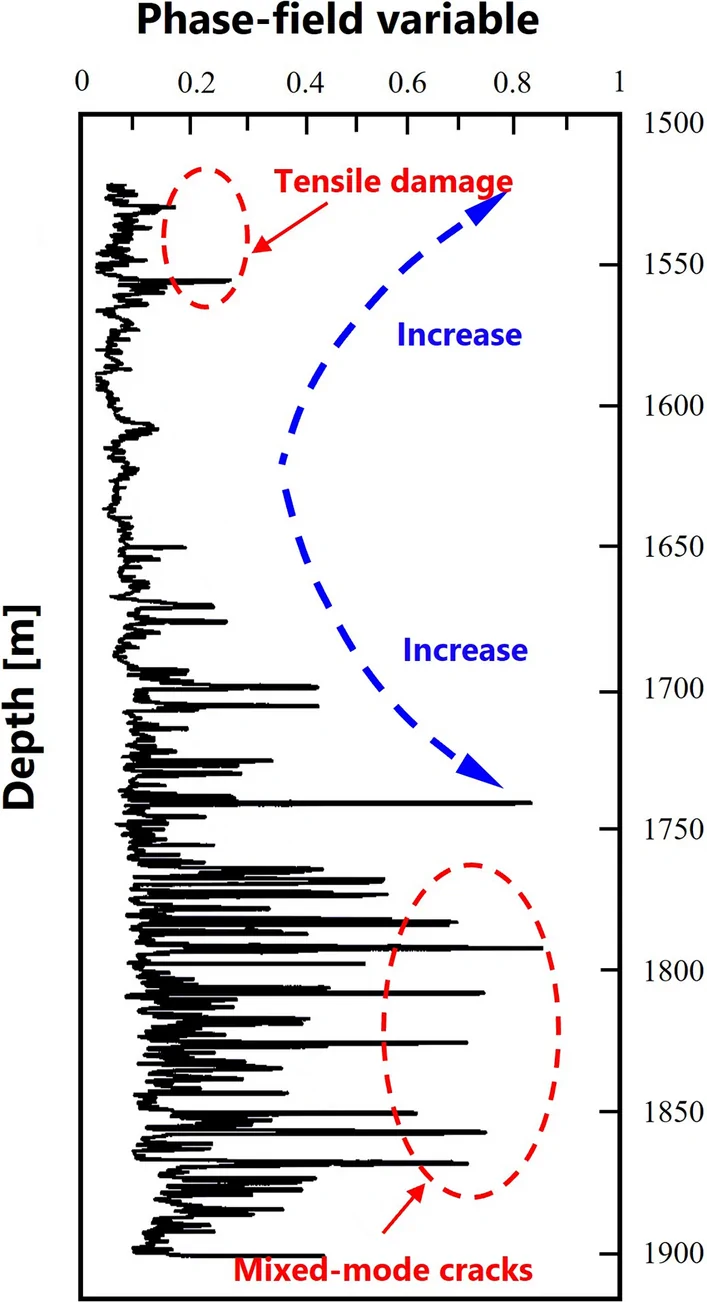
Inversion results of wellbore cement sheath failure from acoustic logging data of a monitored salt cavern gas storage (He et al. 2022a)

To validate and interpret the numerical results, we compared the simulated failure patterns with acoustic logging inversion data from a monitored salt cavern gas storage facility in China that has been in operation for five years (He et al. 2022a). As shown in Fig. 24, the failure index ranges from 0 (intact) to 1 (fully failed). The field data reveal that failure severity increases with depth, with the middle section showing lower levels of deterioration than the wellhead and bottom regions. This trend is consistent with the simulation results presented in this study. Previous investigations of salt cavern wellbores under coupled plasticity and fatigue conditions are also reported similar findings (He et al. 2022a, 2023). Taken together, the strong agreement between simulation and field observations supports the validity and reliability of the proposed gas themo-mechanical coupled phase-field model.

### Discussions

Our findings reveal several useful storage design and operational strategies that are beneficial for enhancing the long-term integrity of the cement sheath within cavern systems. These strategies include adopting the progressive rate-change scheme recommended in our previous research (Liu et al. 2021): sudden changes in injection and production rates should be avoided, with sufficient time allocated for heat transfer and thermal stabilization. Some additional mitigation strategies comprise pre-cooling injection gases or pre-heating produced gases to reduce thermal stress accumulation. Furthermore, enhanced monitoring of the wellbore pressure, temperature, and failure evolution is essential, as is the development of high-performance cement sheath materials (low brittleness and high toughness). Last but not least, attention must be paid to comprehensive safety risk management throughout the entire wellbore depth, as failure modes and mechanisms differ at varying depths.

## Conclusions

In this work, a computational paradigm for gas themo-mechanical coupling in salt cavern gas storage was developed within the phase-field framework. The approach integrates a thermodynamic model of gas flow with a robust phase-field formulation, enabling simultaneous simulation of gas pressure and temperature evolution, as well as deformation and failure of the wellbore cement sheath. The framework has been validated against analytical solutions, laboratory experiments, and field-scale monitoring data, confirming its reliability for both qualitative and quantitative analyses. Based on extensive numerical simulations, the following conclusions are drawn:

- **Gas–thermal response:** the characteristic response time of gas flow is significantly shorter than that of heat transfer. During injection and production, gas pressure exhibits nearly linear variations, while gas temperature evolves nonlinearly with sharp rises or drops followed by short recovery periods. Heat transfer forms localized hot or cold zones of about 0.2 m radius around the wellbore, and temperature distributions along monitoring lines follow a logarithmic relation.
- **Depth-dependent failure:** the failure pattern and severity of the cement sheath vary with wellbore depth. The wellhead region is prone to tensile failure during gas injection, high-pressure storage, and early production, while the bottom of the wellbore is more susceptible to interfacial and mixed-mode failures due to high geostress and complex multiphysical coupling. The simulation results are consistent with acoustic logging inversion data from field monitoring.
- **Thermal cycling effects:** cyclic thermal stresses during injection and production contribute to progressive failure accumulation, although their influence on the overall failure mode is limited. Once failure develops, the reduced thermal conductivity of the cement sheath impedes heat transfer, leading to localized overheating or overcooling near the damaged zones.
- **Material and stress effects**: the lateral stress coefficient $K_\textrm{c}$ exerts a strong influence on failure patterns and critical operating pressures, with the maximum capacity observed at $K_\textrm{c} = 1$. By contrast, fracture energy release rate and elastic modulus have limited effect on failure mode but strongly affect the critical pressure. Higher fracture energy and lower stiffness increase the critical operating pressure, thereby enhancing wellbore integrity.
- **Operational strategy:** rapid injection–production cycling significantly accelerates cement sheath failure under themo-mechanical coupling. A progressive injection–production scheme is recommended to minimize abrupt stress and temperature fluctuations.

This study is one step in our broader effort to understand how underground gas storage wells stay safe when many physical processes act at the same time. We are currently investigating how the cement sheath can crack and debond from the casing inside the wellbore (Fei and Choo 2020b; Wang and Wu 2023; He et al. 2022a). In future work, we will extend our framework to include additional effects that are important for salt-cavern wellbore integrity, such as subsurface biochemical reactions, damage caused by highly diffusive or corrosive gases (e.g., hydrogen, hydrogen sulfide) (Zhu et al. 2023; Sun et al. 2024; Grant et al. 2025), poroelastic effects (Yoshioka et al. 2021) linked to gas leakage, and large-scale three-dimensional simulations. We also plan to compare and calibrate our model predictions against data from laboratory experiments and field monitoring. Together, these efforts will provide a stronger basis for designing and evaluating the long-term stability of subsurface storage systems for petroleum, natural gas, and hydrogen under realistic operating conditions.

## Figures and Tables

**Fig. 25:**
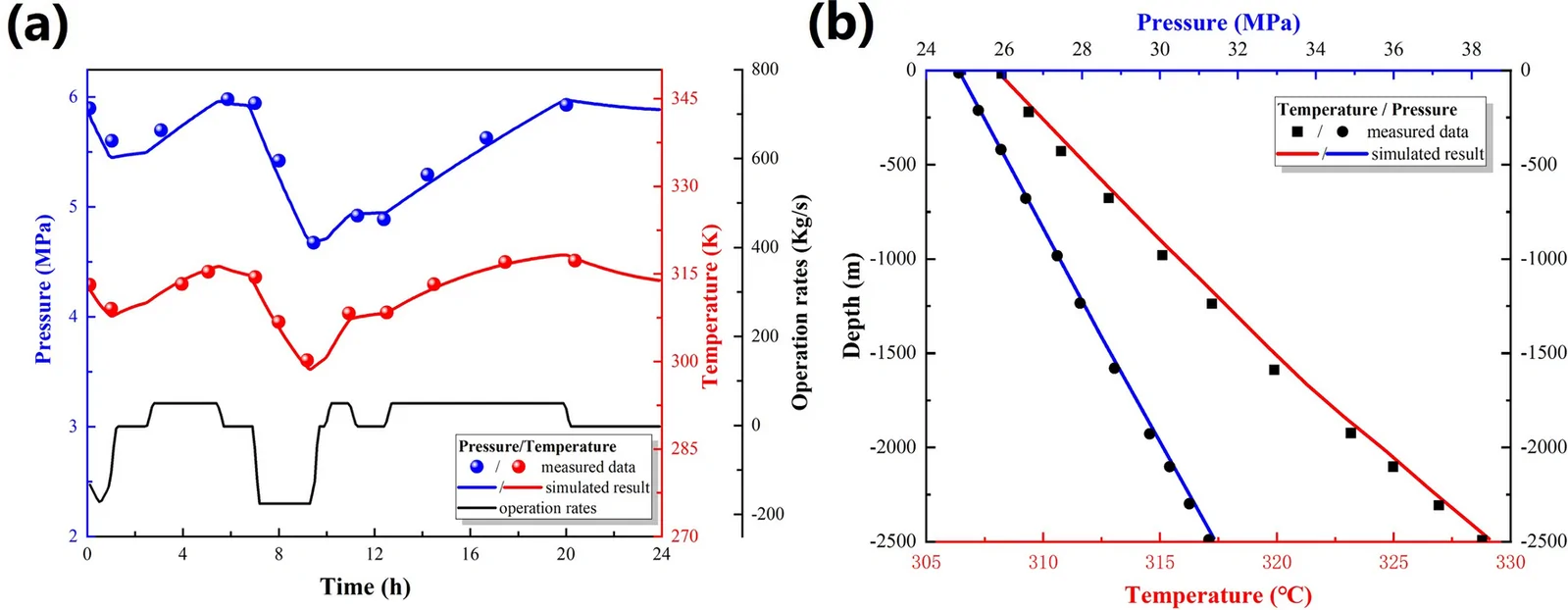
Thermodynamic behavior during gas injection and production. **a** Variations in gas temperature and pressure in the Huntof CAES cavern over a operation day. **b** Simulated temperature and pressure distributions along the wellbore at the Xiangguosi UGS

**Fig. 26:**
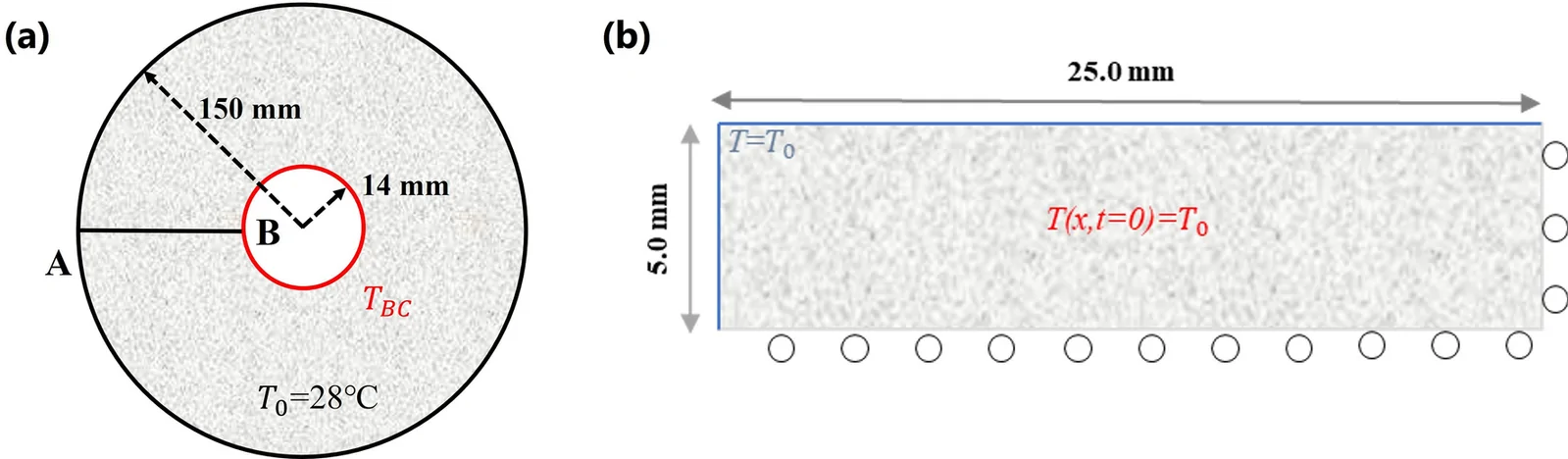
Thermal loading test of the Ishida rock specimen: **a** geometric model and boundary conditions of the heated cylindrical sample; **b** temperature, tangential stress, and radial stress distributions along the A–B cross-section at 94 min

**Fig. 27:**
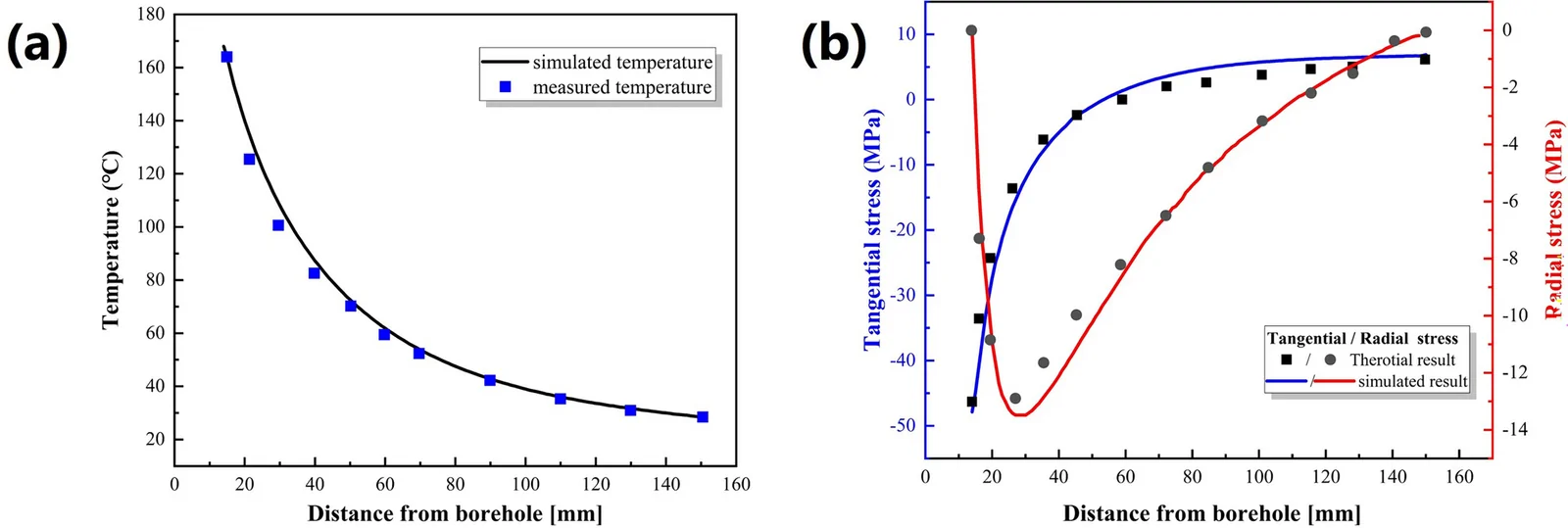
Numerical results of thermo-mechanical coupling process in the heated cylindrical samples: **a** comparison of temperature distribution against the laboratory measurements (Wang et al. 2018b) and **b** comparison of stress distributions against the analytical solutions (Ishida et al. 1990)

**Fig. 28:**
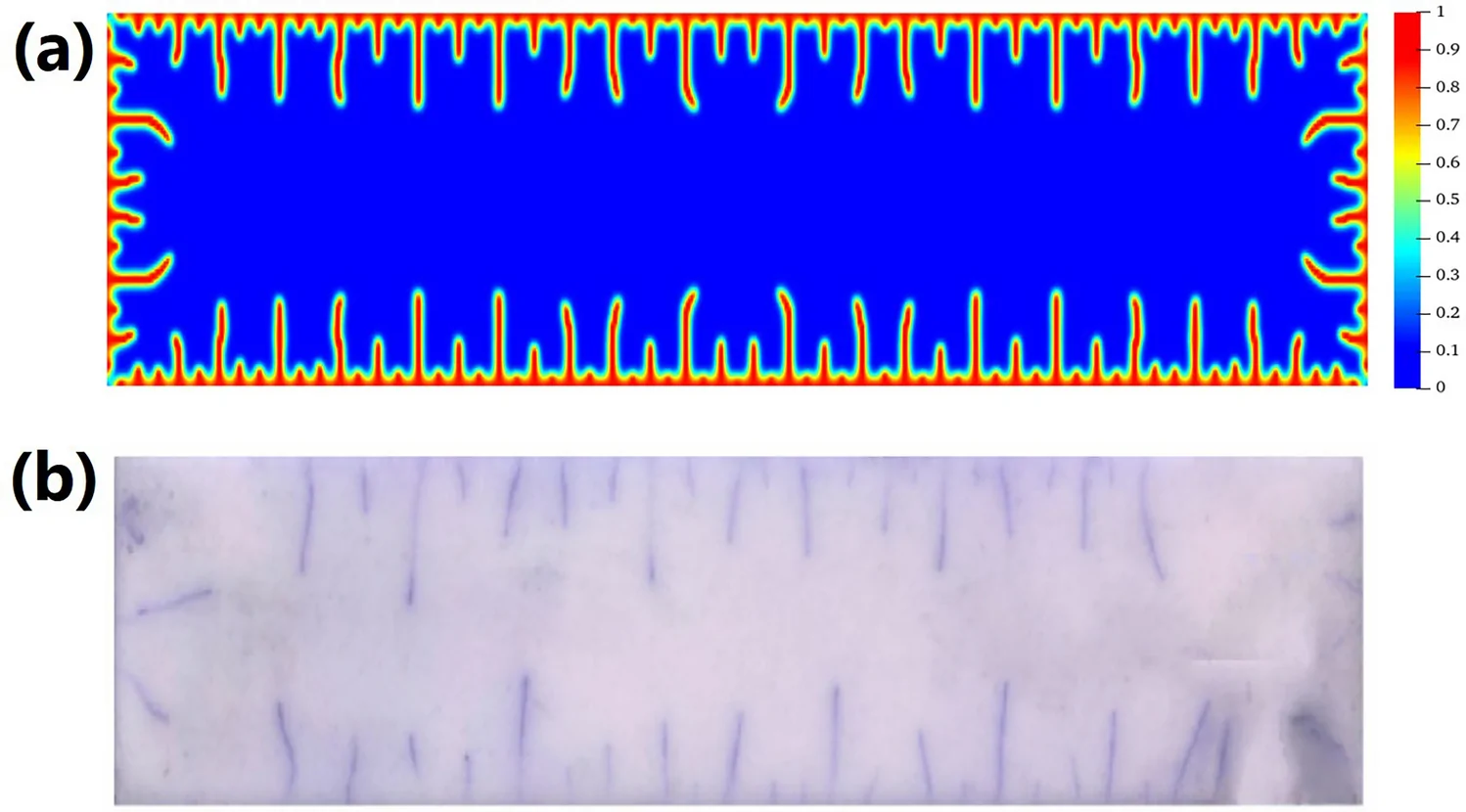
Comparison of the thermal shock crack patterns obtained from **a** the present phase-field simulations and **b** the reported laboratory experiments (Jiang et al. 2012; Wang et al. 2019b)

## Data Availability

Data will be made available on proper request.

## Appendix: Numerical Validation Benchmarks

### Appendix 1: Gas Injection–Production Thermodynamics Model

Due to the lack of complete thermodynamic records for both the salt cavern and the wellbore over entire injection–production cycles, the proposed gas thermodynamic model is validated against two independent datasets: the Huntorf CAES cavern test and the Xiangguosi wellbore measurements (Han et al. 2022). The simulation parameters, summarized in Tables 3 and 4.

Figure 25a presents the simulated evolution of cavern gas temperature and pressure during a single operational day. Both variables exhibit trends consistent with the injection–production sequence: increasing during injection, decreasing during production, and remaining nearly constant during shut-in. During constant-pressure storage, temperature continues to vary due to heat exchange with the surrounding rock mass, leading to secondary pressure fluctuations. The predicted profiles agree well with the Huntorf CAES field data, confirming the robustness of the model at cavern scale.

**Table 3:** Simulation parameters of thermodynamic in the cavern

Parameter	Unit	Values of Huntof
Average cavern radius	m	20
Cavern volume	m^3^	141,000
Cavern surface area	m^2^	25,000
Heat transfer coefficient	W/(m$^2$ K)	30
Initial gas pressure in cavern	MPa	5.9
Initial gas temperature in cavern	K	313.15
Injected gas temperature	K	323.15
Injected gas pressure	MPa	7.2
Injected mass rate	kg/s	50
Withdrawal mass rate	kg/s	190

For wellbore-scale validation, temperature and pressure distributions are calculated under a wellhead boundary condition of 24.8 MPa, 308.15 K, and an injection rate of 3.52 kg/s. As shown in Fig. 25b, pressure increases linearly from the wellhead to the bottom, whereas temperature exhibits a slightly nonlinear rise. The predictions are consistent with field observations from the Xiangguosi site.

Overall, these comparisons demonstrate that the proposed injection–production thermodynamic model can reliably capture the coupled temperature–pressure evolution in both cavern and wellbore environments.

**Table 4:** Simulation parameters of gas flow in wellbore

Parameter	Unit	Values of Xiangguosi UGS
Total length of the wellbore	m	2496.6
Inner radius of the tubing	m	0.049785
Outer radius of the tubing	m	0.05715
Inner radius of the casing	m	0.11025
Outer radius of the casing	m	0.13615
Roughness of tubing	m	$1.5 \times 10^{-5}$
Thermal conductivity of cement annulus	W/(m K)	0.983
Thermal conductivity of protected fluid	W/(m K)	0.6
Thermal conductivity of surrounding rock	W/(m K)	3
Heat transfer coefficient in radiation	W/(m$^2$ K)	100
Geothermal temperature gradient	K/m	0.026
The ground temperature	K	288.15
Injected mass rate	kg/s	3.52
Withdrawal mass rate	kg/s	6.63
Injected gas pressure of wellbore	MPa	24.8
Injected gas temperature of wellbore	K	308.15
Withdrawal gas pressure of wellbore	MPa	28
Withdrawal gas temperature of wellbore	K	363.15

### Appendix 2: Thermo-Mechanical Coupling Model

The capability of the proposed model to compute thermal stress is validated against a benchmark heating experiment conducted by Ishida et al. (1990). In the test, the borehole wall temperature was raised from 28 to 310 $^\circ$C within three hours at a constant heating rate of 1.5 $^\circ$C/min. The rock specimen, shown in Fig. 26a, is a cylindrical sample with a diameter of 150 mm and a central borehole of 28 mm. The thermo-mechanical parameters of the rock are: Young’s modulus E=47 GPa, Poisson’s ratio ν=0.25, thermal conductivity *k* = 2.3 W/(m $^\circ$C), volumetric specific heat capacity $c_v=2.8 \times 10^6$ J/(m$^3$ $^\circ C$), and thermal expansion coefficient $\alpha =0.85 \times 10^{-5}~^\circ \textrm{C}^{-1}$. The initial temperature was $T_0=28\,^\circ$C, while the borehole wall temperature was prescribed as a time-dependent boundary condition, $T_{\textrm{BC}}(t_{\textrm{real}})=28+1.5,t_{\textrm{real}}$ (minutes).

Under these thermo-mechanical loading conditions, an analytical solution for thermal stress (Ishida et al. 1990) is available and is adopted here as the reference for numerical validation.

A-1 $$\begin{aligned} \left\{ \begin{aligned} \sigma _\theta&= \frac{\alpha E}{r^2} \left[ \frac{r^2 + a^2}{b^2 - a^2} \int _a^b rT\, dr + \int _a^r rT\, dr - Tr^2 \right] \\ \sigma _r&= \frac{\alpha E}{r^2} \left[ \frac{r^2 + a^2}{b^2 - a^2} \int _a^b rT\, dr - \int _a^r rT\, dr \right] \end{aligned} \right. \end{aligned}$$

where $\sigma _\theta$ and $\sigma _r$ denote the tangential and radial stresses, respectively; α is the linear thermal expansion coefficient; *E* is the Young’s modulus; *a* and *b* represent the inner and outer diameters of the cylindrical specimen, respectively; *T* is the temperature; and *r* is the radial distance from the borehole center.

Figure 27b–c shows the temperature, tangential stress, and radial stress distributions along the A–B cross-section of the specimen at 94 min. The temperature attains its maximum at the borehole wall and decreases gradually toward the outer boundary. The tangential stress follows a similar spatial trend, but with compressive stresses near the borehole and tensile stresses near the outer boundary. In contrast, the radial stress remains compressive across the entire domain, with the largest magnitude concentrated at the borehole wall. Overall, the results from the proposed thermo-mechanical coupling phase-field model agree well with the experimental temperature measurements and the analytical thermal stress solution.

To further validate the proposed thermo-mechanical coupling phase-field model for failure simulation, the classical ceramic plate quenching experiment is considered (Wang et al. 2019b), which can be recognized as a benchmark. A ceramic plate with dimensions of $50 \times 10 \times 1~\textrm{mm}$ (only one-quarter modeled due to symmetry) is uniformly heated to an initial temperature $T_0$ and then rapidly quenched in a water bath at $20^\circ \textrm{C}$, as depicted in Fig. 26b. The mechanical boundary conditions are imposed by the prescribed horizontal displacement along the right edge and the vertical displacement at the bottom, while thermal symmetry is maintained by insulating both edges. This plate is initially heated to $T_0=300\, ^\circ \textrm{C}$, with Dirichlet boundary conditions of $T=20 \,^\circ \textrm{C}$ applied to the left and right surfaces. The material properties are as follows: Young’s modulus $E_0=370~\textrm{GPa}$, Poisson’s ratio $\nu _0=0.3$, tensile strength $f_t=180~\textrm{MPa}$, fracture energy $G_f=42.47~\mathrm {J/m}^2$, thermal expansion coefficient $\eta _t=7.5 \times 10^{-6}~\textrm{K}^{-1}$, density $\rho =3980~\mathrm {kg/m}^3$, thermal conductivity $\kappa _0=31$ W/(m K), and specific heat capacity c=880 J/(kg K). The numerically predicted crack pattern is presented in Fig. 28a, demonstrating excellent agreement with the experimentally observed fracture morphology (Jiang et al. 2012; Wang et al. 2019b), as plotted in Fig. 28b.
